# Valorization of biochars derived from corn straw waste for the adsorption of methylene blue and methyl orange dyes: influence of acid and alkaline activation

**DOI:** 10.1007/s11356-026-38116-w

**Published:** 2026-08-06

**Authors:** Thalita da Silva Neto, Bianca A. R. Silva, Rennan F. S. Barbosa, Fernanda R. Pinhati, Maria Ismênia S. T. Faria, Derval S. Rosa, Daniella Regina Mulinari

**Affiliations:** 1 Faculdade de Tecnologia, Universidade do Estado do Rio de Janeiro (UERJ), Resende-RJ, Brazil; 2 Centro de Engenharia, Modelagem e Ciências Sociais Aplicadas (CECS), Universidade Federal do ABC (UFABC), Santo André-SP, Brazil; 3 Departamento de Engenharia dos Materiais (DEMAR), Universidade de São Paulo (USP EEL), Lorena-SP, Brazil

**Keywords:** Corn residue, Biochar, Acid activation, Alkaline activation, Dyes adsorption

## Abstract

The increasing utilization of agro-industrial waste underscores the necessity for sustainable valorization strategies consistent with circular economy principles. In this study, corn husk residue was investigated as a precursor to produce chemically activated biochars with tunable surface chemistry and porosity, enabling targeted adsorption of the cationic dye methylene blue (MB) and the anionic dye methyl orange (MO). Two activation strategies, acidic (H₃PO₄) and alkaline (NaOH), were applied to tailor the physicochemical properties of the resulting materials, designated as acid corn biochar (ACB) and alkaline corn biochar (AKCB). ACB exhibited uniform mesoporosity and polar surface groups, which enhanced interactions with both dyes, while AKCB demonstrated hierarchical porosity and neutral or basic surface groups that promoted *π*–*π* interactions and affinity for anionic molecules. Both biochars effectively removed dyes from aqueous solutions; however, ACB achieved higher adsorption capacities, reaching 60.2 mg g⁻^1^ for MB and 54.4 mg g⁻^1^ for MO, compared to 33.7 mg g⁻^1^ and 40.8 mg g⁻^1^ for AKCB, respectively. These findings indicate that chemical activation of corn-based biochar is a promising, cost-effective approach for developing efficient adsorbents for water treatment. This study advances process intensification and circular economy objectives by converting agricultural waste into functional materials for the remediation of industrial effluents.

## Introduction

The generation of agro-industrial waste has increased significantly due to the expansion of agricultural production and the growth of large-scale food processing. Corn, one of the most widely cultivated crops worldwide, produces substantial amounts of residues as byproducts of grain processing. In the 2024/2025 period, global corn production was estimated at around 328.3 million metric tons, with its residue primarily consisting of stalks, leaves, cobs, and husks. Among these, husks account for approximately 50% of the total residues, representing a substantial portion of agricultural waste that is often underutilized (Wang et al. 2022). Given the environmental impacts of improper disposal, this residue is used in several applications, including reinforcement in composites (Mirițoiu et al. 2025), catalytic modification (Huang et al. 2023), corn-based biosensors (Zhang et al. 2023), fuel (Balamurugan et al. 2018), and water treatment.

One of the most promising approaches to adding value to this residue is its conversion into biochar, a carbon-rich material obtained as a byproduct of the thermochemical conversion of biomass under limited oxygen conditions (Ahmad et al. 2014). Biochar has garnered increasing attention in research due to its diverse environmental benefits, including pollution reduction, promotion of a circular economy, and support for sustainable development efforts (Bano et al. 2025). Its inherent characteristics such as surface chemistry, structural properties, large surface area, and high porosity make it an effective adsorbent, which is further enhanced through activation processes (Bano et al. 2025). To improve surface reactivity, different activation methods can be employed, the most common being physical (Yi et al. 2021), thermal (Dziemianowicz et al. 2022), and chemical activation (Kurzweil 2025).

Physical activation can be achieved by purging steam during pyrolysis to remove active carbons, thereby increasing surface area (Xu et al. 2021; Alvarez et al. 2015). Thermal treatment, on the other hand, is a process that enhances the surface area and porosity of biochar through high-temperature treatments, often involving oxidizing agents like steam or carbon dioxide (CO₂). This method is notable for removing volatile compounds and developing a porous structure (Jiang et al. 2024).

As previously explained, the process of physical and thermal activation can reduce the carbon matrix’s resistance to chemical oxidation, increasing its susceptibility to chemical degradation (Xu et al. 2021). Aiming to overcome this drawback, chemical activation can be a suitable alternative. It can be performed using alkaline agents such as potassium hydroxide (KOH) and sodium hydroxide (NaOH), or acidic agents such as phosphoric acid (H₃PO₄), sulfuric acid (H₂SO₄), and zinc chloride (ZnCl₂), resulting in biochar materials with distinct textural and chemical properties (Neme et al. 2022). Regarding chemical activation, it can enhance both thermal and physical properties, resulting in improved chemical and thermal oxidation stability, a higher fraction of stable carbon, and an increase in adsorbable functional groups (Xu et al. 2021; Pathy et al. 2023).

Alkaline activation, typically employing KOH or NaOH, facilitates redox reactions during thermal treatment, removes volatile compounds, and enhances micropore formation within the carbon structure (Kamdem Tamo et al. 2025). NaOH disrupts lignocellulosic bonds and catalyzes selective thermal degradation, thereby yielding a more porous, open carbon matrix. This structure supports the adsorption of small molecules, including metal ions and cationic dyes, due to its negatively charged active sites. The efficiency of alkaline activation depends on operational parameters, including the activating agent ratio, pyrolysis temperature, and reaction time (Pereira et al. 2024).

In contrast, acid activation is commonly performed with phosphoric acid (H₃PO₄) or sulfuric acid (H₂SO₄), primarily altering the chemical composition of the biochar surface. Phosphoric acid functions as a dehydrating agent during pyrolysis, reducing tar formation and promoting the development of condensed aromatic structures (Xu et al. 2021). This process introduces oxygenated functional groups, such as hydroxyl, carboxyl, and phosphate, which increase surface polarity and enhance affinity for anionic pollutants. Although acid activation does not significantly increase surface area, it effectively enhances chemical interactions with contaminants through ion exchange, dipole–dipole interactions, and complexation (Neme et al. 2022).

Both chemical activation methods improve biochars adsorbent performance: alkaline activation enhances porosity, while acid activation enriches surface functional groups. Thus, the choice of activating agent and conditions should align with the target contaminant and intended application. When optimized, these strategies yield biochars capable of efficiently removing organic and inorganic pollutants, including dyes, potentially toxic elements, and pharmaceuticals (Andrade et al. 2020; Xu et al. 2021; Pathy et al. 2023; Bano et al. 2025).

Moreover, combining thermal and chemical activation further improves structural organization, surface area, and adsorption capacity, while enhancing thermal and chemical stability. This integrated approach enables better control of textural and surface properties for specific environmental applications (Premchand et al. 2024).

Environmental remediation with biochar involves its use to remove harmful substances from contaminated soils, water, and air. In liquid effluent treatment, biochar serves as a selective adsorbent, capturing toxic compounds via mechanisms such as pore-filling, electrostatic attraction, and chemical interactions with surface functional groups. In gas purification, biochar can adsorb volatile organic compounds (VOCs) and potentially toxic elements (PTEs) from industrial emissions, reducing air pollution (Bano et al. 2025).

Synthetic dyes, which are widely used in the textile, cosmetic, and food industries, pose significant environmental concerns. Methylene blue and methyl orange exemplify cationic and anionic dyes, respectively, both characterized by high water solubility and resistance to biological degradation, which complicates their removal through conventional treatment processes (Li et al. 2023). In addition to their visual impact, these dyes pose toxicity risks to aquatic organisms and humans. Methylene blue has been associated with neurotoxic and respiratory effects, while methyl orange exhibits potential mutagenic and carcinogenic properties (Radoor et al. 2021). Table 1 presents biochar derived from various biomass feedstocks and the corresponding adsorbed substances.

**Table 1:** Biochar’s derived from various biomass feedstocks and their respective adsorbed substances

*Biomass feedstock*	*Activation method*	*Adsorbed substance*	*Reference*
*Banana peel waste*	Chemical alkaline activation (NaOH) followed by pyrolysis at 600 °C	Methylene blue	(Pereira et al. 2024)
*Eucalyptus*	Pyrolysis followed by chemical acid activation with H_3_PO_4_	Chromium (VI)	(Zeng et al. 2021)
*Rice husk*	Thermal treatment at 800 °C	Methylene blue	(Jiang et al. 2024)
*Corn straw*	Catalytic modification using nanosized Pt/TiO_2_ via low-temperature oxidation process (220 °C)	Cadmium (Cd)	(Huang et al. 2023)
*Coconut shell*	Chemical activation using ZnCl_2_	Potentially toxic elements (Pb^2+^, Fe^2+^, Cu^2+^, Zn^2+^)	(Chai et al. 2021)
*Pomegranate peel*	Chemical alkaline activation with NaOH	Acebutolol (antibiotic)	(Akkari et al. 2023)

Despite considerable progress in developing biochar-based adsorbents, significant knowledge gaps remain. Most published studies have focused on biochars produced via a single activation route or evaluated their performance against only one contaminant class. Consequently, the comparative influence of acidic and alkaline activation on the physicochemical properties of corn straw-derived biochars, as well as the resulting adsorption mechanisms for cationic and anionic dyes, remains poorly understood. Furthermore, few studies have correlated surface chemistry, textural characteristics, and adsorption performance. Bridging these gaps is essential for selecting the most suitable activation strategy and optimizing biochar production for practical wastewater treatment applications.

Based on these considerations, this study investigates the production of corn straw-derived biochars via acidic and alkaline chemical activation and compares their physicochemical characteristics and adsorption performance for methylene blue (MB) and methyl orange (MO). Additionally, response surface methodology (RSM) was employed to optimize adsorption conditions, while equilibrium and kinetic studies were conducted to elucidate the adsorption mechanisms. This approach provides a comprehensive assessment of how different activation strategies influence biochar properties and their applicability as sustainable adsorbents for industrial wastewater treatment.

## Materials and methods

### Materials

Corn residues were collected at the street fair in Lorena, São Paulo, Brazil. They were dried at 80 °C for 48 h to remove moisture. Once the moisture had been removed, the residues were crushed, ground in a mill, and sieved to obtain samples that passed through a 35-mesh screen. The methylene blue (MB) and methyl orange (MO) dyes, sodium hydroxide (NaOH), and phosphoric acid (H_3_PO_4_) reagents were acquired from Dinâmica Química Contemporânea Ltda.

### Acid/basic modification of biochar

The corn biochar prepared by acid modification (ACB) was produced using the following method: the dried corn residue (CR) was impregnated with a 40% m/m aqueous solution of H₃PO₄ at a 1:4 ratio (CR: acid solution/active agent) at room temperature for 24 h (Zeng et al. 2021). Afterward, the sample was dried at 80 °C for 48 h to obtain acid-activated corn fiber (ACF). The ACF was then pyrolyzed at 600 °C for 1.5 h in a muffle furnace under an argon atmosphere, with a gas flow rate of 100 mL min^−1^. Finally, the biochar was washed with distilled water until the pH reached 7, then dried at 90 °C for 24 h, yielding the acid-activated biochar (ACB).

**Fig. 1:**
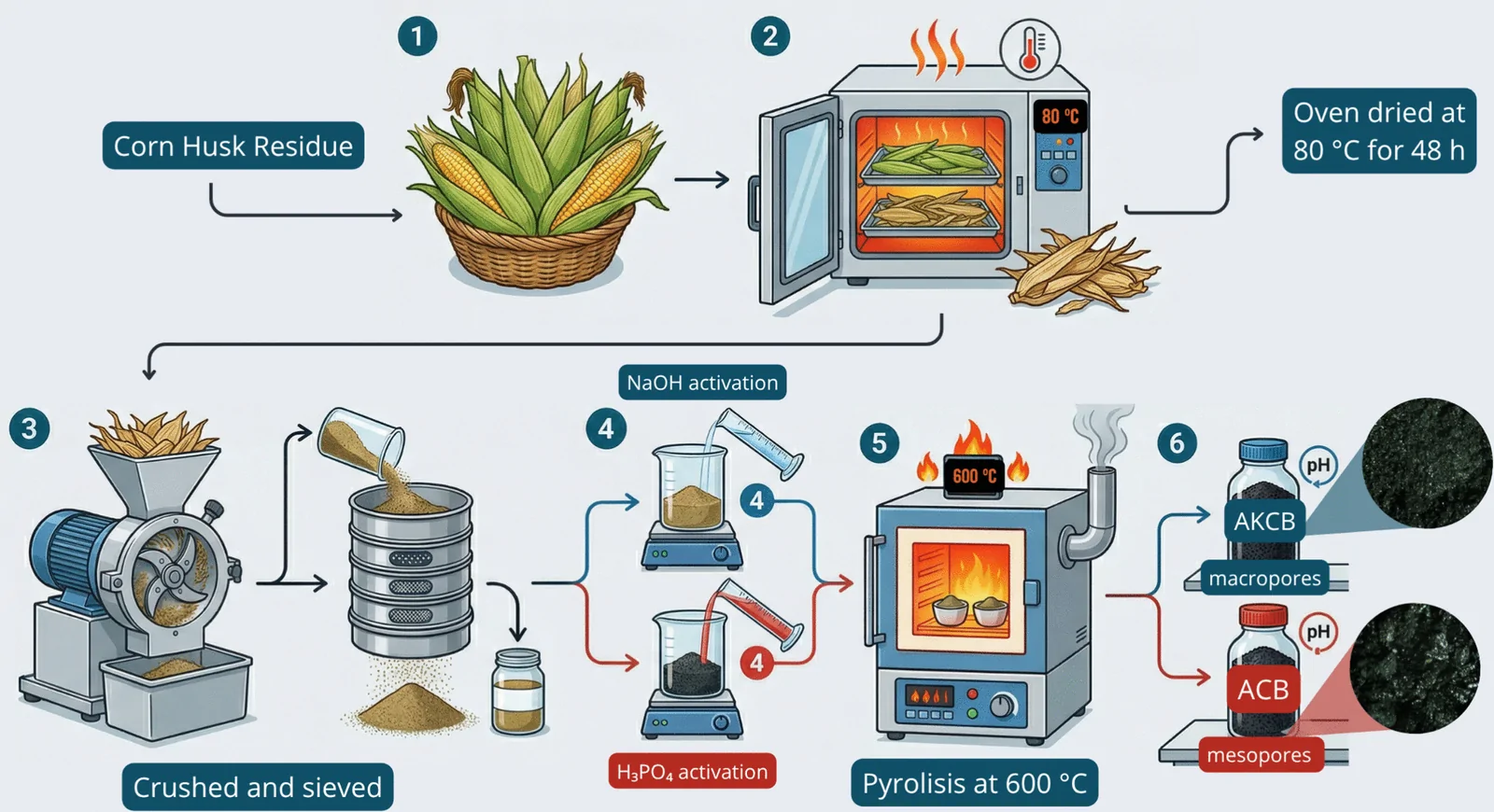
Illustration of the methodology for producing biochar from two methods of utilizing corn residues: acid-activated corn biochar (ACB) and alkaline-activated corn biochar (AKCB)

In the alkaline modification process, the procedure was similar to that of H₃PO₄ activation and followed these steps: the dried corn residue (CR) was treated with an 80 g L^−1^ aqueous NaOH solution at a 1:3 ratio (residue to alkaline solution/active agent) at room temperature for 24 h (Pereira et al. 2024). Subsequently, the sample was dried at 100 °C for 24 h to obtain alkaline-activated corn fiber (AKCF). The AKCF was then pyrolyzed at 600 °C for 1.5 h in a muffle furnace under an argon atmosphere at a flow rate of 100 mL min^−1^. Afterward, the biochar was washed with distilled water until the pH reached 7, then dried at 80 °C for 24 h, yielding alkaline-activated biochar (AKCB). Figure 1 illustrates the methodology for both activation processes.

### Characterization of the materials

Different characterization techniques were employed to determine the distinct physicochemical properties of the corn biochar produced by the two activation methods.

The proximate analysis of the materials was performed to determine moisture and ash content. The moisture content of the samples was measured using a Shimadzu moisture analyzer (Model MOC63u, Shimadzu Corporation, Japan), employing a heating ramp up to a maximum temperature of 160 °C. Elemental composition, including the weight fractions of carbon (C), hydrogen (H), nitrogen (N), and oxygen (O), was assessed using a Flash EA 1112 Elemental Analyzer (Thermo Scientific).

The chemical structures of samples were analyzed using Fourier-transform infrared spectroscopy (FTIR) (Frontier 94942 Model, PerkinElmer Inc., Massachusetts, USA) with an attenuated total reflectance (ATR) diamond accessory. The data was gathered through 64 scans, with a spectral resolution of 4 cm^−1^ in the range of 4000–500 cm^−1^.

The morphological characterization of the materials was performed using scanning electron microscopy (SEM) with a TESCAN Mira 4 FEG-SEM, equipped with a FEG Schottky electron emitter. The samples were fixed onto carbon tape and placed on a suitable sample holder for analysis. Images were acquired using secondary electron (SE) and backscattered electron (BSE) modes, operating at a voltage of 5 keV. The particle size and size distribution were determined using a Mastersizer 3000 analyzer (Malvern Instruments, UK).

The structural characterization of the samples was performed using X-ray diffraction (XRD) with a LabX XRD-600 diffractometer (Shimadzu, Japan) equipped with Cu-Kα radiation (*λ* = 1.54067 Å), operating in the 2θ range of 10 to 40°. This analysis aimed to identify both crystalline and amorphous phases present in the materials.

The thermal stability and decomposition temperature of the samples were analyzed using a TA Instruments SDT Q600 thermogravimetric analyzer. The measurements were conducted under a nitrogen atmosphere (10 mL min^−1^) over a temperature range of 30–600 °C, with a controlled heating rate of 10 °C min^−1^.

Zeta potential (pH_ZPC_) measurements to determine the point of zero charge were performed by dispersing 0.1 g of ACB and AKCB in 50 mL of a NaCl solution (0.1 mol L^−1^). Subsequently, the pH of the suspension was adjusted within the range of 1.0 to 10.0 using solutions of HCl (0.1 mol L^−1^) and NaOH (0.1 mol L^−1^). After 24 h of equilibrium, the final pH values were recorded.

### Adsorption analysis

All adsorption analyses were conducted at a pH of 8.0, based on zeta potential results, the standardization of experimental conditions, and environmental relevance, considering that many real-world effluents are slightly alkaline (Moyo et al. 2023).

#### Design of experiments (DOE)

An experimental design was employed to evaluate the adsorption conditions of the studied materials, focusing on the influence of three key parameters: contact time, dye concentration, and adsorbent dosage. Each factor was tested at three distinct levels: (i) contact times of 10, 60, and 90 min; (ii) dye concentrations of 25, 75, and 150 mg L⁻^1^; and (iii) adsorbent dosages of 2.5, 5.0, and 10 mg mL⁻^1^.

The experimental planning was conducted using Design-Expert® software and employed response surface methodology (RSM), yielding a total of 14 experiments. All tests were performed in triplicate. For each run, a defined mass of adsorbent was added to an Erlenmeyer flask containing 20 mL of dye solution at the predetermined concentration. The flasks were agitated on an orbital shaker at 150 rpm for the specified contact time. After agitation, the suspensions were filtered to remove the adsorbent particles, and the residual dye concentrations were determined by UV–Vis spectrophotometry (Thermo Scientific GENESYS 40, USA) using the maximum absorption wavelengths (*λ*max) of 665 nm for methylene blue (MB) and 430 nm for methyl orange (MO). The absorbance values were converted to dye concentrations using previously established calibration curves, and these concentrations were subsequently used to calculate the adsorption capacity and removal efficiency according to Eqs. (1) and (2):

1 $${q}_{e }= \frac{\left({C}_{0}-{C}_{e}\right)V}{m}$$

2 $$\mathrm{S}\mathrm{o}\mathrm{r}\mathrm{p}\mathrm{t}\mathrm{i}\mathrm{o}\mathrm{n}\;\mathrm{e}\mathrm{f}\mathrm{f}\mathrm{i}\mathrm{c}\mathrm{i}\mathrm{e}\mathrm{n}\mathrm{c}\mathrm{y} \;\left(\%\right)=\frac{\left({C}_{0}-{C}_{e}\right)}{{C}_{0}}\times 100$$

where: ${q}_{e}$ is the amount of dye (mg g^−1^) of adsorbent; *C*_0_ and *C*_*e*_ are the initial concentration of the adsorbate and the concentration of adsorbate in equilibrium with the adsorbent, in mg L^−1^, respectively; V is the volume of the solution; m is the mass of the adsorbent in g.

#### Kinetic study

A kinetic adsorption study was conducted to evaluate the effect of contact time on adsorption capacity at a fixed adsorbate concentration and adsorbent dosage until equilibrium was reached. Based on the results of the optimization experiments using Design-Expert® software, a dosage of 2.5 mg mL⁻^1^ and an initial adsorbate concentration of 150 mg L⁻^1^ were selected. The experiments were performed by agitating the flasks on an orbital shaker at 150 rpm for various contact times (10, 30, 90, 180, 360, 720, 1440, and 2880 min). The amount of adsorbate adsorbed at each time interval (${q}_{e}$) was determined, allowing the construction of the adsorption kinetics curve. The pseudo-first-order model, proposed by Lagergren, is defined by the differential Eq. 3.

3 $$\frac{{d}_{{q}_{t}}}{dt}={k}_{1}\left({q}_{e}-{q}_{t}\right)$$

which, upon integration under appropriate initial conditions, yields the linear form shown in Eq. 4.

4 $$\mathrm{ln}\left({q}_{e}-{q}_{t}\right)=\mathrm{ln}\left({q}_{e}\right)-{k}_{1}t$$

where ${q}_{t}$ is the amount of adsorbate at time *t*; ${q}_{e}$ is the amount adsorbed at equilibrium; ${k}_{1}$ is the rate constant of pseudo-first-order adsorption.

The pseudo-second-order model, developed by Ho and McKay (1999), assumes that the adsorption rate is proportional to the square of the number of unoccupied sites, and is described by Eq. 5.

5 $$\frac{d{q}_{t}}{dt}={k}_{2}{\left({q}_{e}-{q}_{t}\right)}^{2}$$

Its linearized form is demonstrated by Eq. 6.

6 $$\frac{t}{{q}_{t}}=\frac{1}{{k}_{2}{q}_{e}^{2}}+\frac{t}{{q}_{e}}$$

where ${k}_{2}\left(\frac{g}{\mathrm{m}\mathrm{g}.\mathrm{m}\mathrm{i}\mathrm{n}}\right)$ is the pseudo-second-order rate constant.

#### Effect of initial concentrations and isotherms

The study of sorption through adsorption isotherms can provide insights into how MB and MO are adsorbed and estimate the maximum dye uptake capacity of the activated carbon. Accordingly, the samples were exposed to solutions with varying concentrations of MB and MO (75, 150, 225, 300, 375, and 450 mg L⁻^1^) for 10 min in the case of the acidic carbon. For the basic carbon, the MB and MO concentrations used were 25, 50, 75, 100, 125, and 150 mg L⁻^1^ for 30 min. Both sets of samples were stirred at 150 rpm to assess the fit to the isotherm models. The sorption isotherm models applied were the Langmuir, Freundlich, and Temkin models.

#### Langmuir isotherm

One of the most used isotherms for adsorption is the Langmuir isotherm. It considers that the adsorption process occurs in a single layer of molecules on the adsorbent surface, but this process does not take place at the adsorbed sites. The Langmuir can be described by Eq. 7:

7 $${q}_{e}=\frac{{q}_{m}{K}_{L}{C}_{e}}{1+{K}_{L}{C}_{e}}$$

where ${q}_{m}$ is the sorbent monolayer capacity; *K*_*L*_ is the sorption free energy constant; ${C}_{e}$, the equilibrium concentration; ${q}_{e}$ is the equilibrium dye concentration of the sorbent, ${q}_{m}$, and can be determined from linear adjustment using ${C}_{e}/{q}_{e}$ and ${C}_{e}$ data.

#### Freundlich sorption isotherm

As a variation of the Langmuir model, the Freundlich model is an empirical model considering multilayer adsorption, where the surface of the adsorbent has heterogeneous sites. The Freundlich model can be described by Eq. 8:

8 $$qe={K}_{F}{C}_{e}^\frac{1}{n}$$

where ${K}_{F}$ is the sorption capacity constant andn, is the sorption intensity constant.

If n = 1, it is a linear sorption process. If n < 1, it is a chemical process. If n > 1, it is a physical process. *K*_*F*_ and n can be calculated by Eq. 9:

9 $$\mathrm{log}qe=\frac{1}{n}\mathrm{log}{C}_{e}+\mathrm{log}{K}_{F}$$

## Results and discussion

### Moisture content and elemental composition

Table 2 shows the moisture and the amount of C, H, and N in the samples studied. Regarding moisture content, the ACB sample exhibited the highest percentage.

**Table 2:** Elemental analysis results (C, N, H, O), calculated atomic ratios (C/H and C/O), and moisture content (%) for the corn biochar (CR), acid-activated biochar (ACB), and alkali-activated biochar (AKCB)

***Samples***		*CR*	*ACB*	*AKCB*
*Elemental analysis (wt. %)*	C	45.8	28.2	72.6
	N	6.3	0.26	0
	H	8.6	3.6	1.9
	O	39.2	67.9	25.4
*Ratios*	C/H	5.3	7.8	38.2
	C/O	1.2	0.42	2.9
*Moisture content (%)*		8.4	23.8	6.1

This result can be attributed to the activation with phosphoric acid (H₃PO₄), which promotes the formation of oxygen-containing functional groups on the biochar surface, such as hydroxyl (–OH), carboxyl (–COOH), and phosphate esters (C–O–P) (Dechapanya and Khamwichit 2023). These hydrophilic groups enhance the material’s affinity for water (Devault et al. 2025). Another contributing factor may be the presence of residual phosphorus-containing compounds in the biochar after activation and washing. These residues can interact with water molecules, further increasing moisture retention (Xu et al. 2021). In respect of elemental composition, a reduction of nearly 38% in carbon content was observed compared to the raw material. This behavior may be attributed to the removal of volatile matter caused by H₃PO₄ activation, capable of generating compounds with lower mass (for example, CH_4(g)_, CO_2(g)_, and CO_(g)_), which are soluble volatile compounds and break down weakly bound substances present on the biochar surface, primarily during the pyrolysis process. At the same time, this treatment may leave some vestiges of H^+^ and PO_4_^3−^ on the biochar surface (Xu et al. 2021; Dechapanya and Khamwichit 2023).

The alkaline treatment yields a more carbonized, hydrophobic surface with fewer polar functional groups, resulting in lower water affinity and reduced moisture content (Sahoo et al. 2021). This process also increases the carbon content through carbonization, which releases heteroatoms, such as nitrogen and oxygen, as volatile compounds (e.g., NH₃), leaving a rigid carbon framework (Pereira et al. 2024). The proposed reaction mechanism is shown in Eqs. 10–14 (Liu et al. 2021).

10 $$6\;\mathrm{NaOH}+2\mathrm{C}\;\longrightarrow\;2\mathrm{Na}+3\mathrm{H}_{2}+2\mathrm{Na}_{2}\mathrm{CO}_{3}$$

11 $$\mathrm{Na}_{2}\mathrm{CO}_{3}\;\xrightarrow\Delta\;\mathrm{Na}_{2}\mathrm{O}+\mathrm{CO}_{2}$$

12 $$\mathrm{CO}_{2}+\mathrm{C}\;\longrightarrow\;2\mathrm{CO}$$

13 $$\mathrm{Na}_{2}\mathrm{CO}_{3}+\mathrm{C}\;\longrightarrow\;2\mathrm{Na}+3\mathrm{CO}$$

14 $$\mathrm{Na}_2\mathrm{O}+\mathrm{C}\;\longrightarrow\;2\mathrm{Na}+\mathrm{CO}$$

Equations 10–14 indicate that as the reactions progress and the pyrolysis temperature increases, the formation of metallic sodium and carbon monoxide and dioxide is favored. This phenomenon significantly contributes to the development of porosity and the elimination of heteroatoms such as oxygen. Consequently, these transformations result in an enrichment of carbon content in the activated biochar compared to the corn residue (Liu et al. 2021). As shown in Table 2, hydrophobicity increased in both samples, with a more pronounced effect in AKCB. This indirect effect can be attributed to the rise in the C/H ratio compared to the CR, suggesting the development of a more carbonized and hydrophobic surface. Such changes typically result in a more porous structure with a greater tendency toward physisorption (Jiang et al. 2024). This increased hydrophobicity could enhance the interaction of biochar treated in an alkaline medium with more nonpolar substances.

### Fourier-transform infrared spectroscopy (FTIR)

**Fig. 2:**
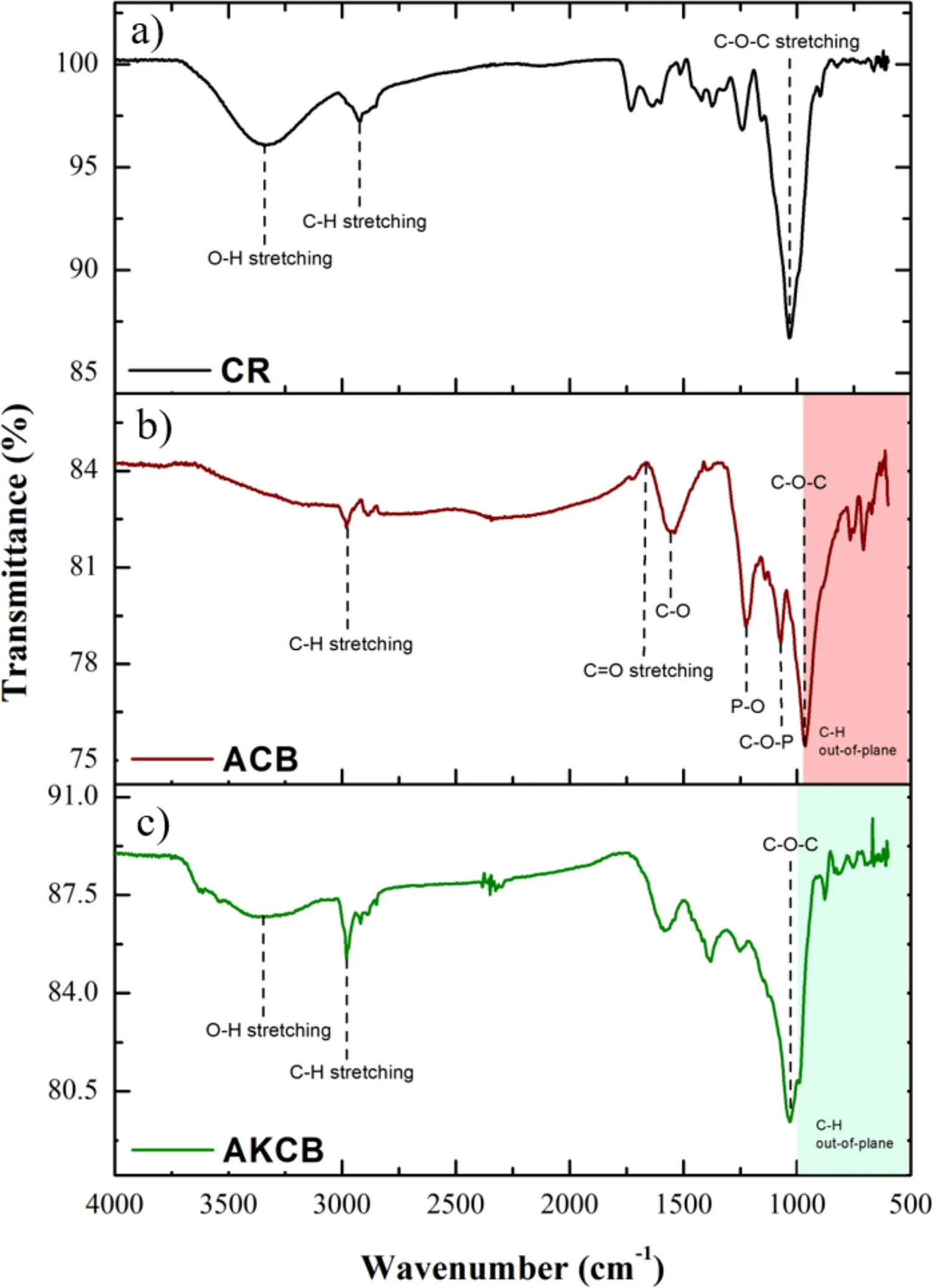
Infrared spectra at 4000–500 cm.^−1^ wavelength obtained from samples. **a** Corn residues (CR). **b** Biochar treated with H_3_PO_4_ acid (ACB). **c** Biochar treated with sodium hydroxide acid (AKCB)

The FTIR spectra of corn residue (CR), acid corn biochar (ACB), and alkaline corn biochar (AKCB) before adsorption tests are shown in Fig. 2a, (b), and (c), respectively.

Considering that corn residues (CR) are primarily composed of cellulose, hemicellulose, and lignin, the FTIR spectra are expected to show characteristic functional groups associated with these components, as shown in Fig. 2a. Broad peaks around 3330 cm⁻^1^ are indicative of O–H stretching vibrations from hydroxyl groups, typically found in alcohols and phenols in cellulose, hemicellulose, and lignin (Jiang et al. 2024). Peaks near 2925 cm⁻^1^ correspond to aliphatic C–H stretching vibrations (Ray et al. 2020), while a prominent peak at approximately 1030 cm⁻^1^ is attributed to C–O–C stretching vibrations of ether linkages in polysaccharides (cellulose, hemicellulose, and lignin) (Wang et al. 2022). After thermal and chemical treatments (as observed in ACB and AKCB), these three peaks showed a significant decrease in intensity, attributed to the decomposition of the biopolymers (Vuong et al. 2023).

The FTIR spectrum of ACB (Fig. 2b) displayed peaks near 1652 cm⁻^1^ and 1349 cm⁻^1^, which can be attributed to the C = O stretching vibrations of carbonyl groups and –CH₃ bending vibrations, respectively. Additionally, in the ACB spectrum, a broad band is observed from 1400 to 1000 cm^–1^, which can be attributed to oxidized carbons and carbons induced by phosphoric acid (Afrooz et al. 2025). This region is essential due to the presence of oxygen-containing functional groups, particularly phosphorus in various forms (including phosphoric esters and P–O bonds). Furthermore, the band observed around 1100 cm⁻^1^ corresponds to C–O–P stretching, indicating the successful formation of phosphate esters following H₃PO₄ acid activation (Dechapanya and Khamwichit 2023). Those acid-oxygenated functional groups can significantly influence the adsorption of dyes by electrostatic interactions and surface complexation with adsorbates (Afrooz et al. 2025). For AKCB (Fig. 2b), the FTIR spectrum suggests a highly carbonized structure with elevated carbon content. This is evidenced by the decrease of the prominent broad O–H stretching band at 3334 cm⁻^1^, implying that oxidation processes were significantly reduced during pyrolysis (Sahoo et al. 2021). At the same time, the continued presence of this broad might contribute to the AKCB’s ability to promote hydrogen bonding with both pollutants (Pereira et al. 2024; Thabede and Shooto 2025). Furthermore, the presence of bands between 1580 and 1600 cm⁻^1^ (C = C stretching) and peaks below 1000 cm⁻^1^ (C–H out-of-plane vibrations) in both ACB and AKCB spectra is typical of aromatic ring structures (Luo et al. 2018). These characteristic vibrations are crucial indicators of a successful aromatization process within the carbon structure, which is essential for effective adsorption.

### Scanning electron microscopy (SEM) and particle size

**Fig. 3:**
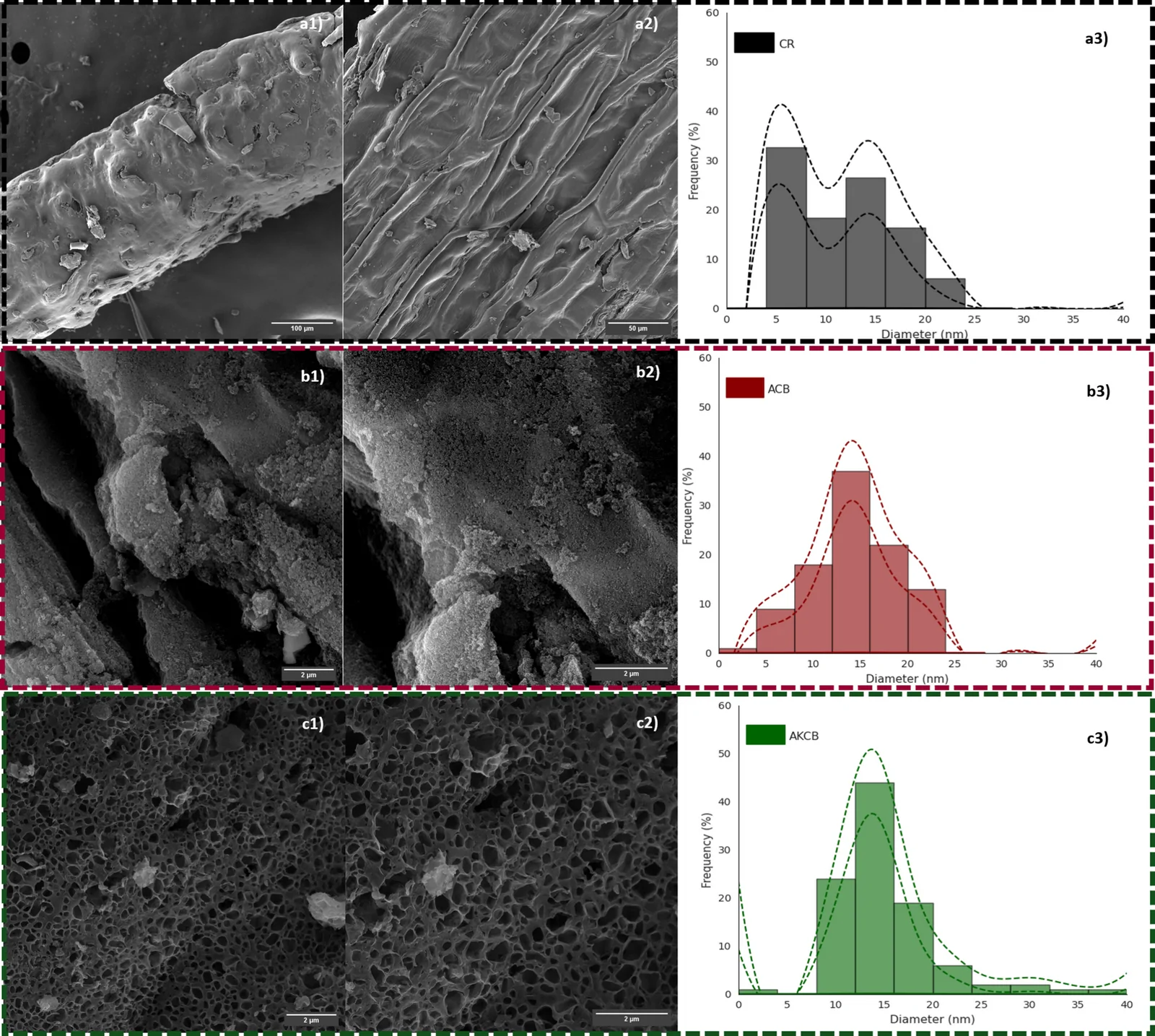
Illustration of the corn residue **a**, samples treated with acidic medium **b**, and alkaline medium **c**. The SEM images of CR, ACB, and AKCB are observed, (**a**1) at 500 × magnification; (**a**2) at 1 k × magnification; (**b**1 and **c**1) at 20 k × magnification; and (**b**2 and c2) at 30 k × magnification. The **a**3, **b**3, and **c**3 represent the particle size histograms of the samples, CR, ACB, and AKCB, respectively

The morphology and particle size of the biochars were analyzed to evaluate the structural changes induced by the different chemical activation agents—phosphoric acid (H₃PO₄) and sodium hydroxide (NaOH)—concerning the raw material (CR), as shown in Fig. 3.

The corn residue (CR) exhibited a smooth, compact appearance and the absence of visible pores, indicating its low initial porosity and the typical fibrous structure of untreated lignocellulosic materials, as shown in Fig. 3 (a1 and a2). This intact morphology may result in low surface area and minimal surface reactivity. It is a characteristic of in natura biomass before thermal or chemical processes, as demonstrated in recent studies on the pretreatment of vegetable waste (Pereira et al. 2024). Duque Sistons et al. (2022) investigated the surface characteristics of coconut fiber under alkaline treatment and observed that untreated fibers exhibited compact, non-porous surfaces with limited adsorption capacity, which significantly improved after chemical activation.

The biochar showed significant surface changes resulting from the action of chemical activators. The activated carbon with H₃PO₄ (ACB) presented a distinct texture, with more defined cavities, the presence of lamellae showing a greater amount of pores, which are evenly distributed, characteristics attributed to the dehydrating promoted by the acid, which leads to the formation of fine pores and partial preservation of the carbonaceous structure, as can be observed in Fig. 3(b2 and b3) (Wang et al. 2023). On the other hand, the activated biochar with NaOH (AKCB) showed disintegration of the original structure, with a rough surface, apparent collapses, and the presence of irregular cavities, even showing different sizes between them, resulting from a more aggressive and selective activation, which promotes the removal of hemicellulose and lignin, creating pores of different sizes (Yu et al. 2024), as shown in Fig. 3(c2 and c3). Similar effects were reported by Sousa et al. 2022, who studied the activation of sugarcane bagasse with NaOH and observed severe surface rupture and irregular porosity, directly linked to enhanced accessibility for adsorption processes.

The morphological differences observed between the activated materials are directly reflected in their textural characteristics, including particle distributions and pore sizes. Particle size analysis indicated that the CR material has a bimodal distribution, typical of untreated biomass (Fig. 3(a3)). The activated materials presented more concentrated distributions, with larger average particle diameters in AKCB and smaller ones in ACB, suggesting differences in the degree of fragmentation and surface structural reorganization, as shown in Fig. 3(b3 and c3).

**Fig. 4:**
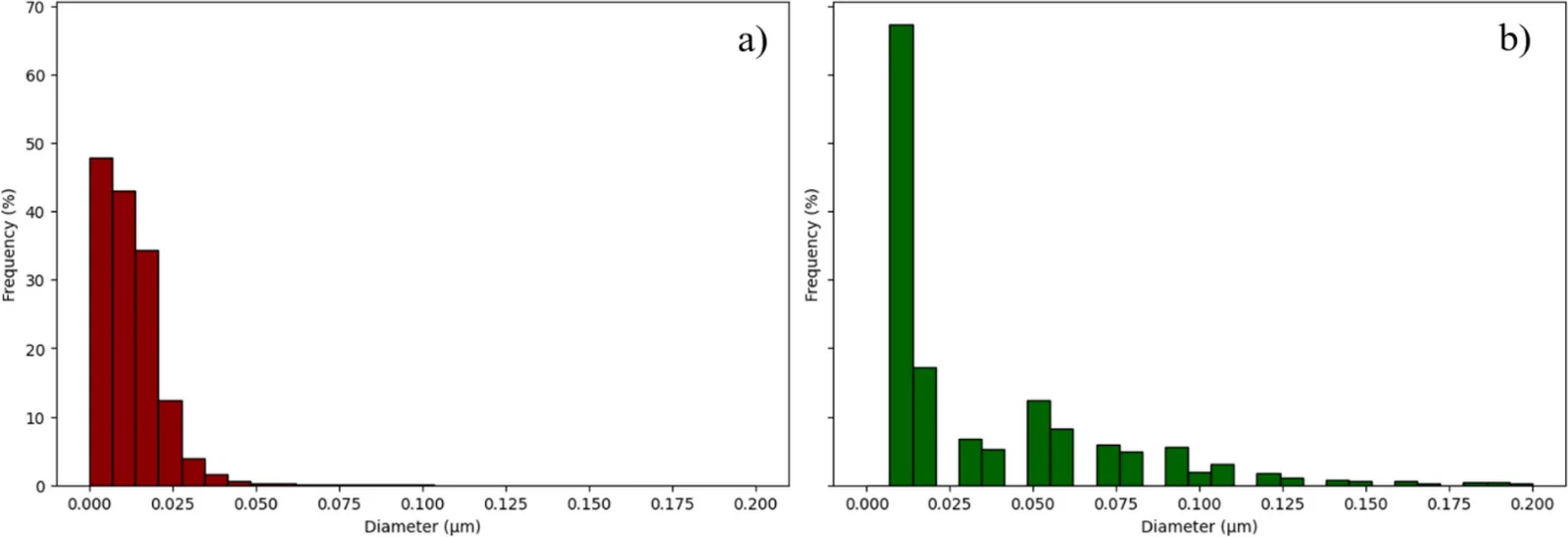
Pore diameter distributions and SEM micrographs of acid-activated carbon (ACB) **a** and base-activated carbon (AKCB) **b**

In terms of porosity, both activated materials developed micro- and mesopores, as measured via ImageJ analysis, with distinct variations depending on the activation method, as shown in Fig. 4. ACB showed a more concentrated distribution of pores between 2 and 20 nm, with a higher frequency in pores below 15 nm, indicating moderately uniform mesoporosity. On the other hand, AKCB revealed a broader distribution, including a significant fraction of pores with diameters greater than 50 nm, indicating a considerable contribution of macropores, as shown in Fig. 4.

These structural differences directly influence dye adsorption. Mesopores facilitate the diffusion of larger molecules, whereas micropores increase the available surface area. Furthermore, functional groups introduced by various activation methods regulate adsorbent–dye interactions through electrostatic forces, *π*–*π* interactions, and hydrogen bonding (Alvarez et al. 2015).

Notably, both types of chemical activation resulted in significant improvements in the morphology, texture, and surface functionality of the materials compared to the original biomass. The structural differences observed between AKCB and ACB resulted in complementary properties. The texture of activated carbonaceous materials represents the set of physical characteristics related to their porous structure, including specific surface area, total pore volume, pore size distribution, and accessibility of internal sites (Wang et al. 2022). These parameters are fundamental for adsorptive efficiency, as they affect both the retention capacity and the kinetics of interaction with contaminants (Wang et al. 2023). Surface functionality is a critical attribute of activated carbonaceous materials, defined by the set of chemical groups located on the external surface and internal pore walls, which modulate properties such as polarity, acid–base character, chemical interaction capacity, and adsorptive selectivity. These groups, including carboxyls, phenols, lactones, ethers, and quinones, are formed under conditions set by the activating agent, the environment, and the process temperature (Sousa et al. 2022).

Oxidized carbonaceous surfaces, for example, exhibit a higher proportion of acidic groups after acid treatment, which directly influences their adsorptive performance (Sousa et al. 2022). Acid-activated carbon (ACB) is characterized by a high level of functionalization, including acidic oxygenated groups. These groups not only increase surface acidity and polarity but also favor adsorption through hydrogen bonding, ion exchange, and complexation with metal cations and polar organic compounds (Chai et al. 2021; Sousa et al. 2022). This behavior is frequently observed after acid modification with acidic conditions, which introduces abundant carboxylic groups and improves selectivity for polar species (Wang et al. 2024). Furthermore, studies have shown that the presence of these groups facilitates the removal of potentially toxic elements (PTEs) via complexation and ion-exchange mechanisms (Ahmad et al. 2014).

In alkaline-activated carbon (AKCB), functionalization tends to be dominated by neutral or basic groups as well as sites with a graphical character and higher electron density (Chen and Wu 2004; Pereira et al. 2024). Alkaline activation preferentially removes carboxylic groups and promotes the formation of electron-rich sites, which favor the adsorption of anionic species or neutral organic molecules through *π*–*π* and induced dipole interactions. These characteristics are further enhanced by alkaline modifications, which expand the porous structure and increase surface basicity (Chen and Wu 2004).

These differences in surface functionalization between ACB and AKCB result in complementary physicochemical properties. ACB, with its acidic and polar functionality, is ideal for the selective adsorption of polar and metallic species in aqueous media, while AKCB, with a less polar surface and functionalized with basic and *π*-active sites, tends to have greater affinity for aromatic or slightly polar organic compounds, in addition to having potential as a catalytic support (Jiang et al. 2024). This complementarity is corroborated by studies that relate the type of surface functionalization to the adsorptive capacity and the selective profile obtained after different chemical treatments (Chen and Wu 2004).

#### X-ray diffraction (XRD)

**Fig. 5:**
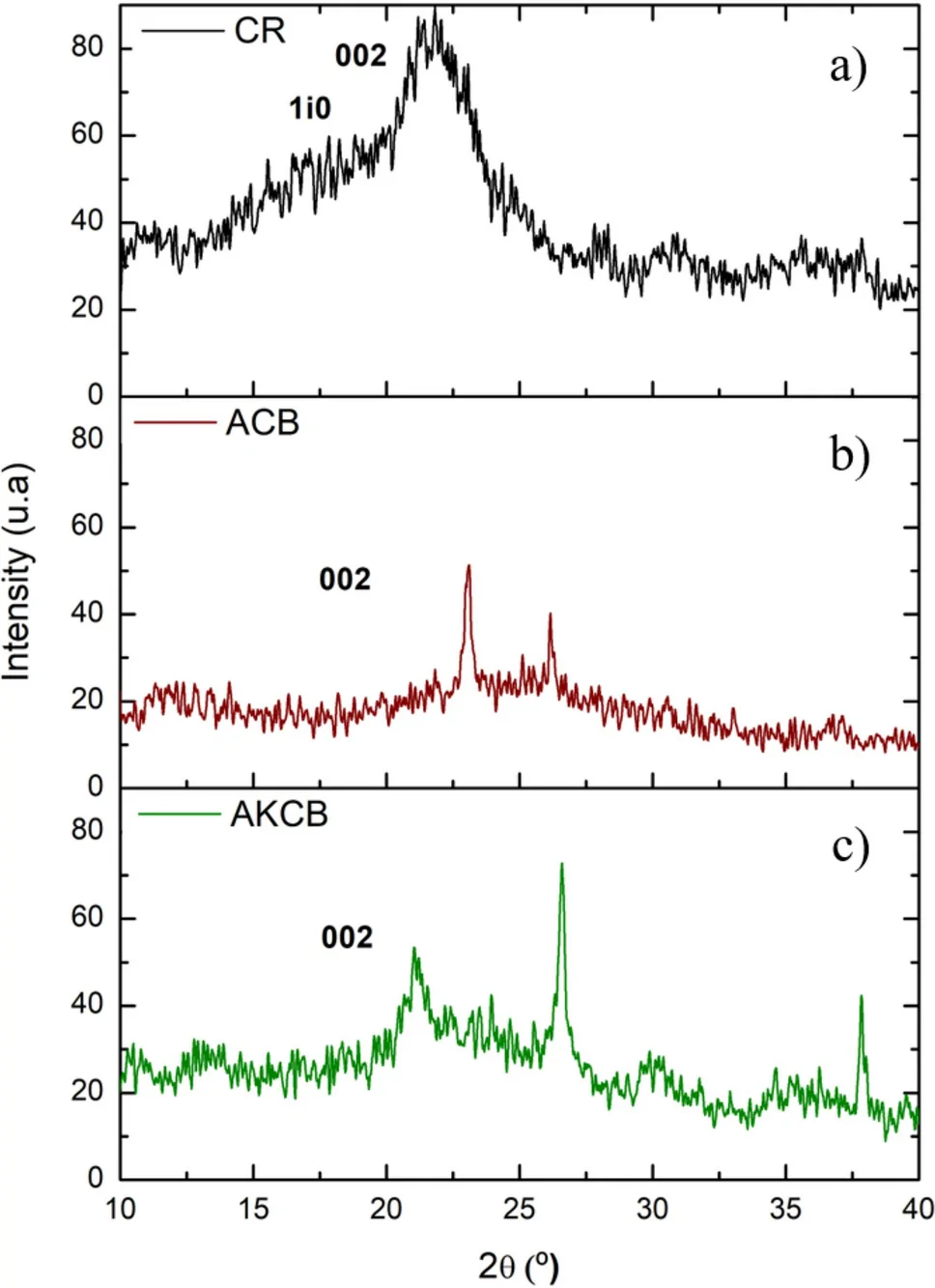
XRD obtained from samples. **a** Corn residues (CR). **b** Biochar treated with H_3_PO_4_ acid (ACB). **c** Biochar treated with sodium hydroxide acid (AKCB)

X-ray diffractometry (XRD) was used to analyze the crystalline structure and organization of the samples (CR, ACB, and AKCB). The results are shown in Fig. 5. The differences observed in the diffraction patterns reflect variations in crystallinity, which indicate differences in the arrangement of the carbon layers and the presence of amorphous or distinct structural features.

Observing the corn residue (CR) diffractogram, it is possible to notice a broad and intense halo centered around 2 θ = 20–25° in the X-axis, with a prominent peak within this halo and a smaller shoulder around 2 θ = 15. This halo is a characteristic of lignocellulosic biomass materials, where the main peak localized at 2 θ = 22 and the shoulder, localized at approximately 15.2° (2θ), are referred to cellulose type I and correspond to the overlapping peaks of the crystallographic planes (002) and (1ī0), respectively (Pereira et al. 2024). Simultaneously, the overall breadth of the halo also demonstrates the presence of amorphous fractions of the biomass, such as hemicellulose and lignin (Wang et al. 2024).

After both acid (ACB) and alkaline (AKCB) activation, the diffractograms revealed a reduction in crystalline peaks, more prominently in the ACB sample. For ACB, two key peaks are observed at 2*θ* = 23.1°, associated with the (002) plane of amorphous carbon, and at 2*θ* = 26.1°, corresponding to the (002) crystalline plane of graphite, mainly related to monoatomic carbon layers organized in microcrystals (Xu et al. 2021). In contrast, the AKCB diffractogram retains a broader amorphous carbon halo and displays a sharper peak in the 25–40° (2*θ*) region, likely linked to residual NaOH. These more defined peaks may result from the formation of new crystalline inorganic phases formed by reactions between NaOH and ash components of corn straw (Wang et al. 2024), including sodium silicates (Na₂SiO₃ and Na₄SiO₃) and sodium carbonates (Na₂CO₃), likely produced from interactions with silica and atmospheric CO₂ during the activation/pyrolysis process (Wood et al. 2024).

The almost complete disappearance of cellulose diffraction peaks in ACB confirms the efficiency of the pyrolysis and acid activation processes, which led to effective depolymerization and carbonization of the original biomass structure, reinforcing the amorphous character of the resulting biochar (Wang et al. 2024). This inherently disordered structure lacks a rigid, repeating crystalline lattice, thereby promoting the formation of an interconnected porous network with a high internal surface area. Consequently, the increased number of incomplete chemical bonds within the matrix contributes to structural imperfections typical of amorphous materials (Li et al. 2025). These features favor the diffusion of contaminants through the adsorbent matrix, while the resulting defects and surface functional groups serve as active adsorption sites (Abdelaziz Lekrine et al. 2024).

#### Thermogravimetric analysis (TGA)

**Fig. 6:**
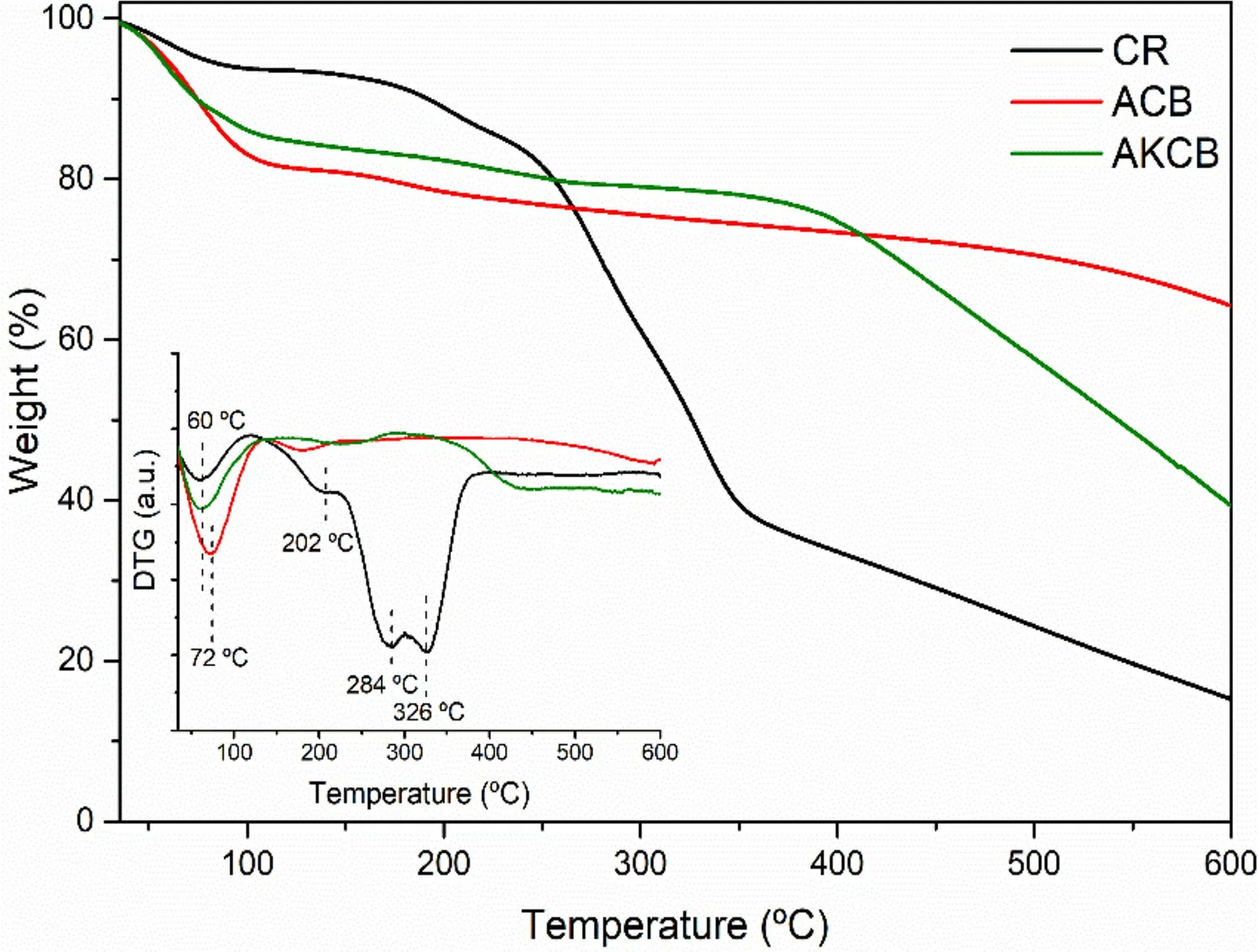
Thermogravimetric curves of corn residue (CR) and the derived biochar using acid route (ACB) and basic route (AKCB), with insertion of the derivative curve (DTG), with main peak temperatures highlighted

 The thermal behavior of corn residue and its derived biochar was investigated, and the resulting thermogravimetric curves are presented in Fig. 6, with the derivative curve shown in the center. The obtained curve shows that the CR sample exhibits lower thermal stability, presenting an initial weight loss at temperatures below 100 °C, as highlighted in the DTG curve, which features a centered weight loss event at 60 °C. In this initial event, the CR sample lost 6.5% of its weight, attributed to a small amount of water and volatile substances. Subsequently, the sample exhibited a series of combined events above 150 °C, with maximum weight loss temperatures of 202, 284, and 326 °C, as indicated in the DTG curve, which are associated with the thermal decomposition of lignin, cellulose, and hemicellulose (Xia et al. 2024). After the degradation of the natural polymers present in the CR, a residual weight of 14.9% at 600 °C was observed, which is associated with inorganic matter that does not degrade within this temperature range (Arwenyo et al. 2024).

It was observed that during the initial event (30–120 °C), the acid and alkaline samples exhibited higher weight losses of 19.7% and 15.2%, respectively. These results may be attributed to the higher surface exposure of the biochar after treatment and the presence of polar groups at its surface. It is noted that the acid sample, which had the highest oxygen content, as observed in the elemental analysis, exhibited greater weight loss, which could be attributed to its enhanced interaction with water via hydrogen bonding. Upon evaluating higher temperatures, the biochar samples exhibit greater thermal stability than the CR sample, without showing a maximum degradation peak in the DTG curve. Nevertheless, the AKCB presented a weight loss associated with the thermo-oxidative degradation of biomass, which may be related to the decomposition of lignin and inorganic materials (Bento et al. 2025). The ACB sample, on the other hand, exhibited lower weight loss, as indicated by the residual weight at 600 °C of 62.7% and 36.3% for ACB and AKCB, respectively, suggesting that the acid treatment promotes a more thermally stable biochar structure (Arwenyo et al. 2024).

#### Determination of the zero-charge point (ZPC)

**Fig. 7:**
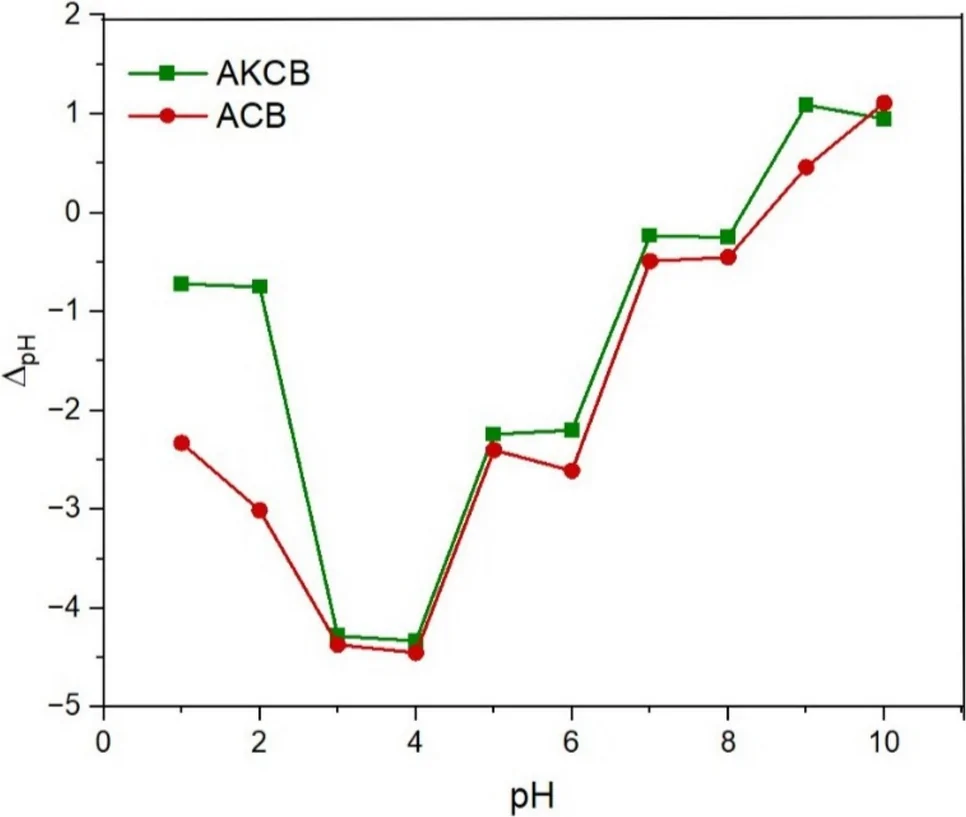
Variation of ΔpH (ΔpH = pH_final − pH_initial) as a function of initial pH for acid-activated biochar (ACB in red) and base-activated biochar (AKCB in green) used to determine the point of zero charge pH_ZPC_

The point of zero charge (pH_ZPC_) is the pH at which the adsorbent surface has zero net charge and plays a key role in adsorption mechanisms involving ionic species. Surface charge behavior varied with the activation method, as illustrated in Fig. 7. Alkaline-activated biochar (AKCB) demonstrated minimal pH variation within the acidic range (pH 2–4), denoting a predominantly negatively charged surface and a high pH_ZPC_, likely greater than or equal to 8. In contrast, acid-activated biochar (ACB) exhibited more pronounced pH shifts, attributed to the introduction of phosphate and hydroxyl groups by H₃PO₄ activation, resulting in a lower pH_pzc_, estimated between 4 and 6.

These findings are consistent with previous observations by Bento et al. 2025, who reported a pH_ZPC_ of around 7 for pinecone residues used for Cr⁶⁺ adsorption. As with pinecone residue, the pH_ZPC_ of the biochars assessed in this study governs electrostatic interactions with charged dye species.

Below the pH_ZPC_, the surface is positively charged, favoring the adsorption of anionic dyes, such as methyl orange (MO). In contrast, above the pH_ZPC_, cationic dye adsorption, such as methylene blue (MB), is promoted.

For all adsorption experiments, a pH of 8.0 was set based on the pH_ZPC_ determination and standardized experimental conditions, allowing a direct comparative analysis between the different activation methods and dye classes. Based on recent literature reports, a wide pH range was tested for cationic and anionic dyes, with alkaline pH showing greater effectiveness for cationic dyes, such as methylene blue (Li et al. 2023; Pereira et al. 2024). However, the adsorption mechanism differs based on the solute’s nature and the activation method. At pH = 8.0, the ACB surface will be predominantly negatively charged. This negative charge density is consistent with the abundant presence of deprotonated acidic groups introduced by the H_3_PO_4_ activation. This favors strong electrostatic attraction and chemisorption with the cationic MB dye (Pereira et al. 2024).

The more basic character of AKCB, attributed to the deprotonation of surface hydroxyls and the removal of acidic functionalities during NaOH activation, enhances its suitability for removing cationic pollutants under neutral to basic conditions. Meanwhile, the acidic surface of ACB with a higher density of polar groups improves interactions with anionic species through mechanisms such as hydrogen bonding and ligand exchange. These complementary properties expand the applicability of both materials in multicomponent dye removal systems (Wang et al. 2023).

### Adsorption analysis

**Fig. 8:**
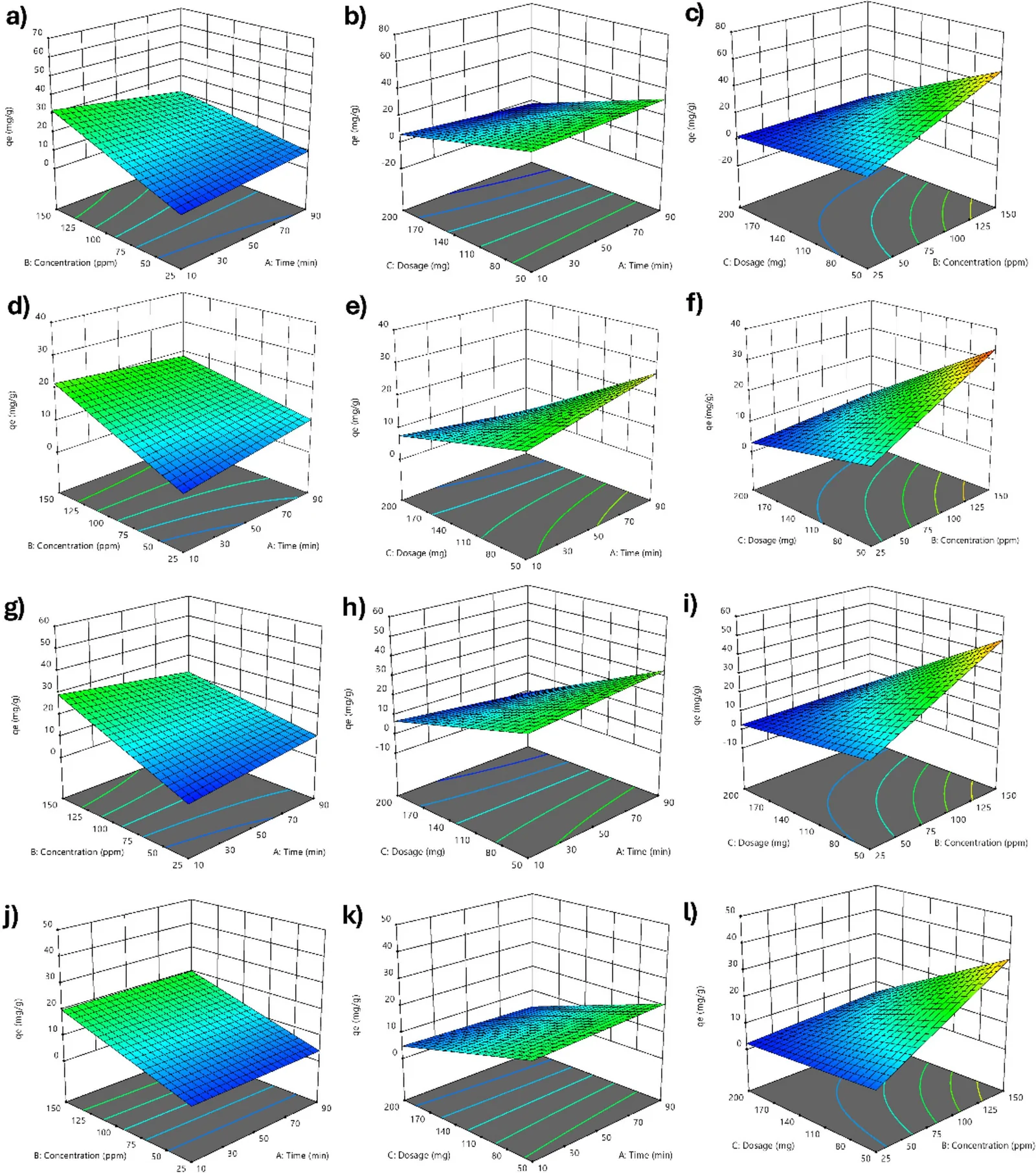
Response surface curves illustrating the effect of adsorption variables on q_e_ for **a**, **b** and **c** (ACB) for methylene blue; **d**, **e**, and **f** (AKCB) for methylene blue; **g**, **h**, and **i** (ACB) for methyl orange; and **j**, **k**, and **l** (AKCB) for methyl orange. All experiments were conducted at the conditions of (i) contact times of 10, 60, and 90 min; (ii) dye concentrations of 25, 75, and 150 mg L⁻^1^; and (iii) adsorbent dosages of 2.5, 5.0, and 10 mg mL⁻^1^ and pH = 8.0 for both dyes

An experimental design based on response surface methodology was applied to assess the combined effects of contact time, dye concentration, and adsorbent dosage on biochar adsorption of methylene blue and methyl orange. The data were fitted to a 2FI model and evaluated by ANOVA (Tables 3, 4, 5, and 6), with response surfaces presented in Fig. 8.

**Table 3:** ANOVA 2FI model adjusted parameters for ACB to remove methylene blue, where highlighted cells indicate significative parameters with *p* value lower than 0.05

	Sum of squares	df	Mean square	*F* value	*p* value
Model	4401.10	6	733.52	16.32	8.53E-04
A-Time	16.06	1	16.06	0.36	5.69E-01
B-Concentration	496.38	1	496.38	11.04	1.27E-02
C-Dosage	1051.23	1	1051.23	23.39	1.89E-03
AB	73.12	1	73.12	1.63	2.43E-01
AC	30.73	1	30.73	0.68	4.36E-01
BC	513.19	1	513.19	11.42	1.18E-02
Residual	314.60	7	44.94

**Table 4:** ANOVA 2FI model adjusted parameters for AKCB to remove methylene blue, where highlighted cells indicate significative parameters with *p* value lower than 0.05

	Sum of squares	df	Mean square	*F* value	*p* value
Model	1529.36	6	254.89	33.16	8.49E-05
A-Time	5.39	1	5.39	0.70	4.30E-01
B-Concentration	220.97	1	220.97	28.75	1.05E-03
C-Dosage	371.42	1	371.42	48.32	2.21E-04
AB	24.83	1	24.83	3.23	1.15E-01
AC	50.72	1	50.72	6.60	3.71E-02
BC	104.41	1	104.41	13.58	7.80E-03
Residual	53.80	7	7.69

**Table 5:** ANOVA 2FI model adjusted parameters for ACB to remove methyl orange, where highlighted cells indicate significative parameters with *p* value lower than 0.05

	Sum of squares	df	Mean square	*F* value	*p* value
Model	3593.19	6	598.87	19.44	4.89E-04
A-Time	0.26	1	0.26	0.01	9.29E-01
B-Concentration	455.86	1	455.86	14.79	6.32E-03
C-Dosage	846.62	1	846.62	27.48	1.20E-03
AB	52.55	1	52.55	1.71	2.33E-01
AC	51.59	1	51.59	1.67	2.37E-01
BC	400.54	1	400.54	13.00	8.68E-03
Residual	215.69	7	30.81

**Table 6:** ANOVA 2FI model adjusted parameters for AKCB to remove methyl orange, where highlighted cells indicate significative parameters with *p* value lower than 0.05

	Sum of squares	df	Mean square	*F* value	*p* value
Model	1627.19	6	271.20	11.57	4.45E-03
A-Time	0.17	1	0.17	0.01	9.35E-01
B-Concentration	314.93	1	314.93	13.44	1.05E-02
C-Dosage	295.30	1	295.30	12.60	1.21E-02
AB	0.00	1	0.00	0.00	9.90E-01
AC	3.31	1	3.31	0.14	7.20E-01
BC	192.69	1	192.69	8.22	2.85E-02
Residual	140.63	6	23.44

Evaluating the combined effect of contact time and dye concentration in Fig. 8a, d, g, and j, it is possible to observe a similar tendency of slightly increased sorption for both dyes with the increase of time. However, the surface response inclination suggests that shorter interaction times may be sufficient to promote effective interaction between the biochar and the dye. This observation is corroborated by the ANOVA tables for each biochar, which indicates that time is not a significant variable in the model, as shown in Tables 3, 4, 5, and 6. According to Hua and co-workers, the active surface of biochar enables faster adsorption, allowing the material to reach equilibrium with a lower contact time (Ho et al. 1999). Regarding the effect of dye concentration, there is a significant increase in sorption capacity with increasing concentration. This effect was similar for both dyes investigated, indicating that the developed biochar did not show saturation at lower concentrations. As the dye concentration increased, the material could remove higher concentrations of dye, reflected in increased biochar sorption capacity. The ANOVA Tables 3, 4, 5, and 6 confirm this result, indicating that concentration is a significant variable in removing both dyes for both acid and basic biochar. Nevertheless, it was possible to notice in the response surface that higher *q*_*e*_ values were attained for ACB, in the removal of methylene blue and methyl orange, compared to AKCB.

In evaluating Fig. 8b, e, h, and k, which illustrates the effect of contact time and dosage, it is corroborated that contact time does not significantly influence sorption capacity. In contrast, the dosage effect had a considerable impact on sorption properties. In the case of dosage, it was observed that the ACB and AKCB samples had higher sorption capacity values at lower dosages, which was associated with the inversely proportional relationship between the *q*_*e*_ value and the adsorbent mass. ANOVA results presented in Tables 3, 4, 5, and 6 confirm the significant effect of dosage, with a *p* value < 0.05. Xu and co-workers also observed that increasing the adsorbent dose may reduce sorption capacity, since not all available sites are saturated (Xu et al. 2021). It should also be noted that the results indicate that lower dosages are economically attractive for obtaining a high-performance material.

Figure 8 c, f, i, and l present the effect of dye concentration and the dosage effect. These variables had the greatest impact on sorption capacity, as corroborated by the shape of the response surface curves obtained. Additionally, the ANOVA results from Tables 3, 4, 5, and 6 indicate that the isolated variables are significant in the adsorption process, and the combined effect also presents a significant influence. The results suggest that the combination of higher dye concentrations (250 ppm) and lower biochar dosages (0.05 g) provides the best sorption capacity for the developed materials. In evaluating the *q*_*e*_ values, it was observed that the acid biochar showed superior removal, reaching 60.2 mg g^−1^ for methylene blue and 54.4 mg g^−1^ for methyl orange, compared to the basic biochar, which showed 33.7 and 40.8 mg g^−1^ for methylene blue and methyl orange, respectively.

#### Kinetic study

**Fig. 9:**
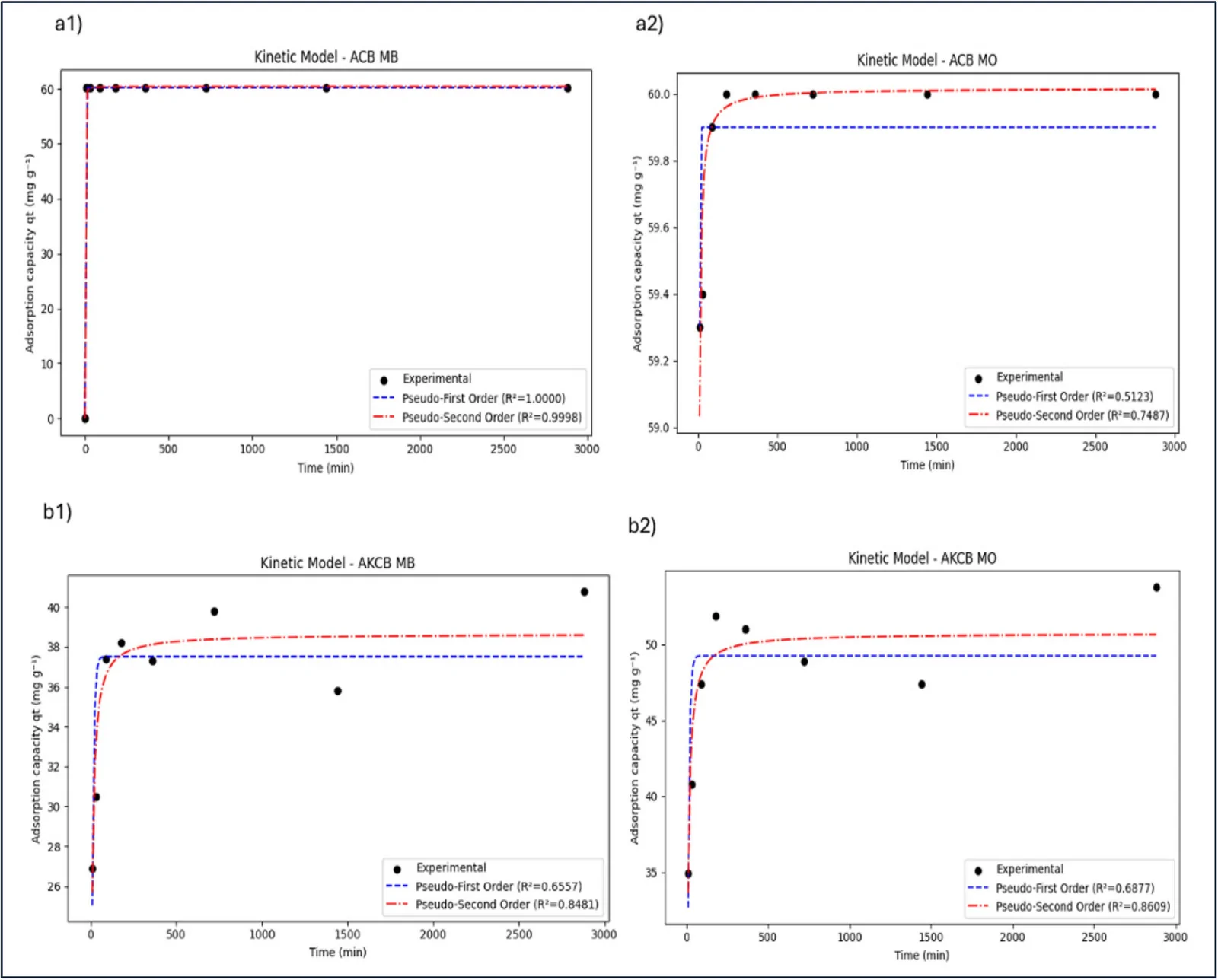
Kinetic model fitting for ACB and AKCB systems: **a**₁ and **a**₂ represent the fittings for MB and MO adsorption onto ACB, respectively; **b**₁ and **b**₂ correspond to the fittings for MB and MO adsorption onto AKCB, respectively. Based on the results of the optimization experiments using Design-Expert® software, the study was conducted with a dosage of 2.5 mg mL⁻^1^ and an initial adsorbate concentration of 150 mg L⁻^1^ for all dye samples. The experiments were performed by agitating the flasks on an orbital shaker at 150 rpm for various contact times (10, 30, 90, 180, 360, 720, 1440, and 2880 min) at pH = 8.0

The kinetic evaluation of the adsorption process, as shown in Fig. 9, was conducted to understand the interaction mechanisms between dyes and activated carbons—a fundamental step in assessing the performance of adsorbents. In this context, the experimental data for the adsorption of methyl orange (MO) and methylene blue (MB) onto activated carbons ACB and AKCB were fitted to the pseudo-first-order (PFO) and pseudo-second-order (PSO) models, as illustrated in Fig. 9.

In general, the PSO model provided a better fit, particularly for ACB systems, suggesting that chemisorption is the primary mechanism. This process entails stronger interactions, such as covalent bonding, between the oxygenated functional groups on the adsorbent surface and dye molecules (Hassaan et al. 2023).

Conversely, the PFO model, typically associated with physisorption, underestimated the adsorption capacities, particularly in AKCB systems. This suggests a multistep sorption process, possibly involving both surface binding and intraparticle diffusion. In the case of MB adsorption onto AKCB, a fast initial uptake followed by a slower approach to equilibrium was observed, indicating the coexistence of different mass transfer mechanisms.

Although adsorption kinetics were not the focus of this study, kinetic modeling was crucial for clarifying and comparing the performance of the chemically modified adsorbents. The better fit of the pseudo-second-order (PSO) model for both ACB and AKCB suggests that chemisorption predominates in the adsorption process. This behavior is consistent with the incorporation of oxygenated functional groups and the surface chemistry modifications promoted by acid and alkaline treatments, which enhance adsorptive interactions (Li et al. 2023).

ACB showed higher adsorption capacity than AKCB, mainly due to the greater density of acidic functional groups (e.g., carboxylic and phenolic) introduced by acid modification, which promote stronger interactions via hydrogen bonding and electron donor–acceptor mechanisms (Bano et al. 2025; Wang et al. 2023). Moreover, acid treatment improves the pore size distribution and increases the accessible surface area (Xu et al. 2021; Alvarez et al. 2015). The synergistic effect of enhanced surface chemical and improved textural properties results in a significantly augmented adsorption performance for ACB.

Collectively, these findings underscore that the superior adsorptive capacity of ACB is not solely due to the quantity of introduced functional groups but also their chemical nature and spatial distribution, which are critical in defining the affinity and selectivity toward the target adsorbates (Xu et al. 2021). This is in line with recent literature demonstrating that the adsorption efficiency of chemically modified activated carbons depends on a careful balance between surface chemistry modifications and pore size optimization. For example, activation with H₃PO₄ resulted in an adsorption capacity for methylene blue, a cationic dye, that was three times higher than that of AKCB. This can be attributed to the acid treatment’s ability to introduce functional groups that interact with adsorbates, along with the greater presence of mesopores in ACB, which enhances both the accessibility and strength of adsorbate–adsorbent interactions (Alvarez et al. 2015). Therefore, the acid treatment approach offers a strategic advantage for tailoring activated carbons with improved performance in adsorption applications, underscoring its potential to advance pollutant removal technologies (Wang et al. 2023).

#### Effect of initial concentrations and isotherms

**Fig. 10:**
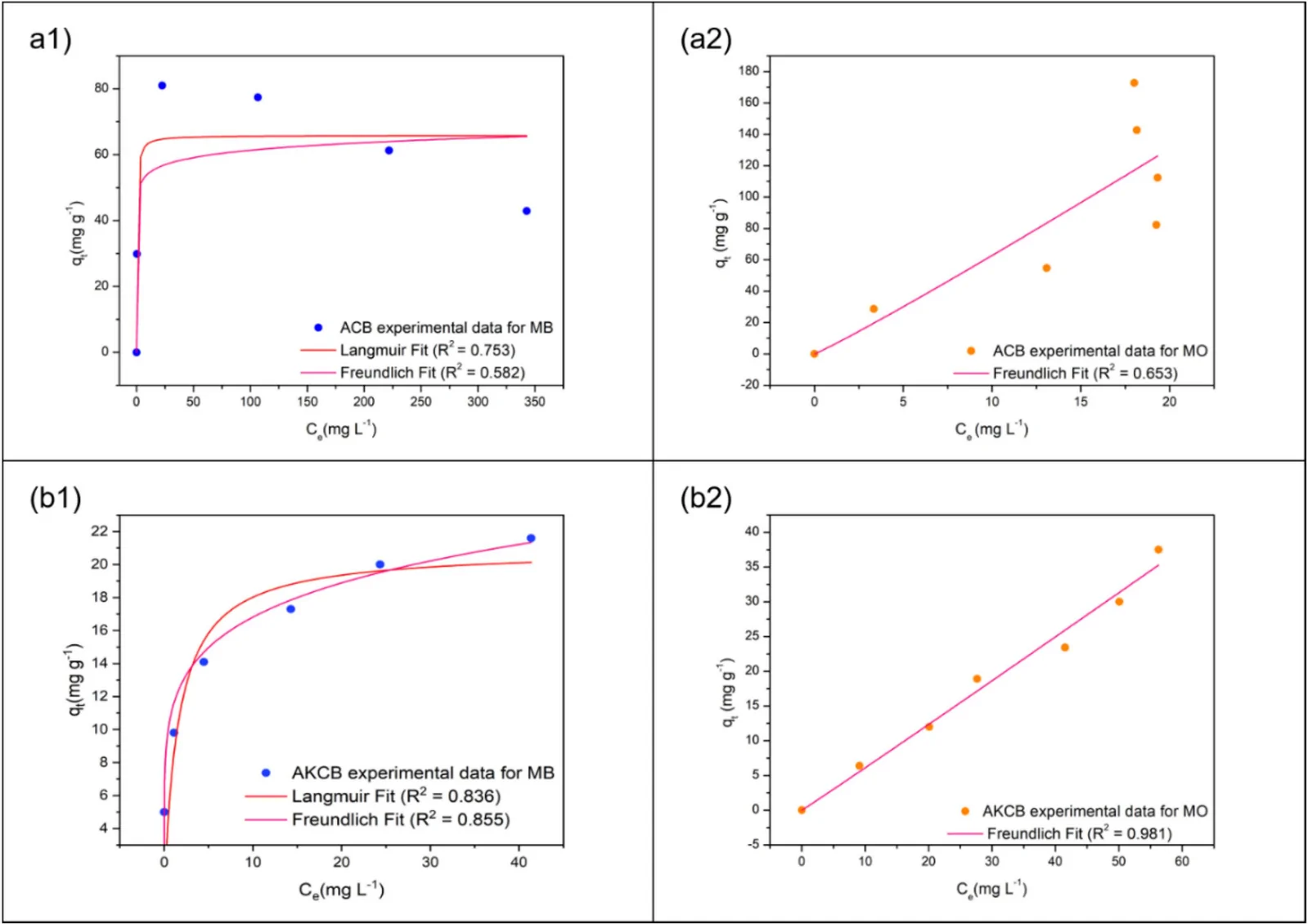
Adsorption isotherms of methylene blue (MB) and methyl orange (MO) dyes onto biochar samples ACB and AKCB. Experimental data were fitted to Langmuir and/or Freundlich isotherm models. **a**1 MB adsorption onto ACB. **a**2 MO adsorption onto ACB. **b**1 MB adsorption onto AKCB. **b**2 MO adsorption onto AKCB

Figure 10 and Table 7 present the Langmuir and Freundlich adsorption isotherm fits for each sample, along with the corresponding isotherm parameters. Adsorption isotherms are fundamental for understanding how adsorbate molecules are distributed between the surface and the liquid phase once equilibrium is achieved. The Langmuir model assumes that adsorption occurs on a homogeneous surface, where the adsorbate forms a monolayer that saturates the pores. Furthermore, regarding the Freundlich adjustment for all MO samples, while a parameter 1/*n* < 1 *(p* parameter) suggests cooperative adsorption (Wang et al. 2024), a value of 1/*n* > 1 indicates robust adsorption resulting from attractive forces between the adsorbent layers (Al-Asadi et al. 2025).

**Table 7:** Estimated MB and MO dyes parameters of kinetic models and Langmuir and Freundlich isotherm models, with the two different biochar samples (ACB and AKCB)

Model	Parameters	ACB (MB)	ACB (MO)	AKCB (MB)	AKCB (MO)
Langmuir isotherm	*q*_máx (_mg g^−1^)	65.8 ± 7.83	-	20.9 ± 2.16	-
	*K*_*L*_ (L mg^−1^)	2.62 ± 2.55	-	0.623 ± 0.356	-
	*R*^2^	0.753	-	0.836	-
Freundlich isotherm	*K*_*F*_ (mg g^−1^)	48.0 ± 16.0	5.46 ± 13.1	11.4 ± 0.0938	0.589 ± 0.232
	*p (1/n)*	18.7 ± 25.0	0.943 ± 0.749	5.96 ± 0.0956	0.985 ± 0.101
	*R*^2^	0.582	0.653	0.855	0.981
Pseudo-first-order	*K* (min^−1^)	0.1	0.12	0.01	0.122
	*q*_e_ (mg g^−1^)	24.82	17.69	26.63	18.29
	*R*^2^	1.000	0.512	0.656	0.688
Pseudo-second-order	*K* (g mg^−1^ min^−1^)	26.52	0.01	0.006	0.01
	*q* (mg g^−1^)	26.52	17.69	28.41	19.43
	*R*^2^	0.999	0.745	0.848	0.861

Regarding methylene blue (MB) adsorption, ACB exhibited a relatively good Langmuir fit (*R*^2^ = 0.753), which can be partially attributed to this model. The maximum adsorption capacity (*q*_max_) was 65.8 mg g^−1^, while KL was 2.62 mg g^−1^ (Fig. 10a1). For Freundlich, the adjustment was not reliable (*R*^2^ = 0.582). In contrast, AKCB presented a satisfactory fit for both Langmuir (*R*^2^ = 0.836) and Freundlich (*R*^2^ = 0.855) models. The Freundlich parameter (1/*n* < 1) further indicates a favorable adsorption mechanism on a heterogeneous surface (Wang et al. 2024). For methyl orange (MO), only AKCB showed a good fit to the Freundlich model, with a 1/*n* parameter < 1 and *R*^2^ = 0.981. This excellent fit suggests a heterogeneous adsorption mechanism driven by strong attractive forces between the adsorbate layers. For methyl orange (MO), only AKCB presented a good fit for the Freundlich model, with a 1/*n* parameter lower than 1 and a *R*^2^ = 0.981. It suggests a heterogeneous adsorption-site mechanism driven by strong attractive forces between the adsorbate layers (Wang et al. 2024). This behavior corroborates the material’s ability to adsorb anionic dyes, a mechanism that likely involves hydrogen bonding, a favorable pH, and pore filling (Pereira et al. 2024; Li et al. 2023). Otherwise, none of the experimental data fits the Temkin model for any of the studied systems. This suggests that the heat of adsorption does not decrease linearly with surface coverage, or that the model’s assumptions do not adequately capture the interactions between the dyes and the biochar surfaces. Table 7 shows the estimated isotherms and kinetic parameters for both MB and MO dyes.

## Conclusion

This study demonstrates that biochars produced from corn residues via distinct chemical activation methods serve as effective and versatile adsorbents for removing both cationic and anionic dyes from aqueous solutions. Acid activation with phosphoric acid yielded a biochar (ACB) with superior adsorption performance, achieving maximum adsorption capacities of 60.2 mg g⁻^1^ for methylene blue and 54.4 mg g⁻^1^ for methyl orange. This enhanced performance is attributed to the introduction of a high density of oxygen-containing acidic functional groups, such as carboxyl and phosphate esters, which increase surface acidity, polarity, and mesoporosity. At neutral pH, the predominantly negatively charged surface of ACB promotes strong electrostatic interactions and chemisorption with methylene blue, while hydrogen bonding and *π*–*π* interactions are significant in the adsorption of methyl orange. In contrast, alkaline activation with NaOH produces a biochar (AKCB) with a more carbonized and hydrophobic surface, characterized by fewer polar functional groups and a pH_ZPC_ near 8. Although AKCB exhibits lower adsorption capacities than ACB, it remains effective for dye removal, achieving 33.7 mg g⁻^1^ for methylene blue and 40.8 mg g⁻^1^ for methyl orange. The adsorption behavior of AKCB is primarily governed by electrostatic attraction toward anionic species and by *π*–*π* and hydrophobic interactions, which enhance its affinity for methyl orange. These results confirm that chemical activation is an efficient strategy for tailoring the surface chemistry and pore structure of corn-residue-derived biochars, enabling selective and complementary adsorption mechanisms. Acid-activated biochar is suitable for the simultaneous removal of cationic and anionic dyes, while alkaline-activated biochar is effective for hydrophobic anionic dyes. These findings underscore the strong potential of chemically activated corn residues as low-cost, sustainable, and tunable adsorbents for industrial wastewater treatment, reinforcing their relevance within circular economy and waste valorization frameworks.

## Data Availability

Data will be available upon request.

## References

[CR1] Afrooz M, Zeynali R, Soltan J, McPhedran KN (2025) A novel biochar adsorbent for treatment of perfluorooctanoic acid (PFOA) contaminated water: exploring batch and dynamic adsorption behavior. J Water Process Eng 69:106586. 10.1016/j.jwpe.2024.106586

[CR2] Ahmad M, Rajapaksha AU, Lim JE, Zhang M, Bolan N, Mohan D, Vithanage M, Lee SS, Ok YS (2014) Biochar as a sorbent for contaminant management in soil and water: a review. Chemosphere 99:19–33. 10.1016/j.chemosphere.2013.10.071 24289982 10.1016/j.chemosphere.2013.10.071

[CR3] Akkari I, Graba Z, Pazos M, Bezzi N, Manseri A, Derkaoui K, Kaci MM (2023) NaOH-activated pomegranate peel hydrochar: preparation, characterization and improved acebutolol adsorption. Water Air Soil Pollut 234:705. 10.1007/s11270-023-06723-9

[CR4] Al-Asadi ST, Mussa ZH, Al-Qaim FF, Kamyab H, Al-Saedi HFS, Deyab IF, Kadhim NJ (2025) A comprehensive review of methylene blue dye adsorption on activated carbon from edible fruit seeds: a case study on kinetics and adsorption models. Carbon Trends 20:100507. 10.1016/j.cartre.2025.100507

[CR5] Alvarez J, Lopez G, Amutio M, Bilbao J, Olazar M (2015) Physical activation of rice husk pyrolysis char for the production of high surface area activated carbons. Ind Eng Chem Res 54:7241–7250. 10.1021/acs.iecr.5b01589

[CR6] Andrade M, Monteiro A, Gontijo E, Bueno C, Macedo J, Rangel E, Melo D, Montero J, Rosa A (2020) Combined analytical Py-GC/MS, SEM, FTIR and ^13^C NMR for investigating the removal of trace metals from aqueous solutions by biochar. J Braz Chem Soc. 10.21577/0103-5053.20200038

[CR7] Arwenyo B, Rodrigo PM, Olabode OA, Abeysinghe HP, Tisdale JN, Azuba RC, Mlsna TE (2024) Comparison of acid- and base-modified biochar derived from Douglas fir for removal of copper (II) from wastewater. Separations 11:78. 10.3390/separations11030078

[CR8] Balamurugan T, Arun A, Sathishkumar GB (2018) Biodiesel derived from corn oil – a fuel substitute for diesel. Renew Sustain Energy Rev 94:772–778. 10.1016/j.rser.2018.06.048

[CR9] Bano A, Aziz MK, Prasad B, Ravi R, Shah MP, Lins PVDS, Meili L, Prasad KS (2025) The multifaceted power of biochar: a review on its role in pollution control, sustainable agriculture, and circular economy. Environ Chem Ecotoxicol 7:286–304. 10.1016/j.enceco.2025.01.004

[CR10] Bento JGGS, Senra LF, Maia LS, Almeida LS, Ferreira LM, Faria MIST, Rosa DS, Mulinari DR (2025) Prediction of Cr6+ removal on the biosorbent from pine cone residue with machine learning simulations. Surf Interfaces 65:106460. 10.1016/j.surfin.2025.106460

[CR11] Chai WS, Cheun JY, Kumar PS, Mubashir M, Majeed Z, Banat F, Ho SH, Show PL (2021) A review on conventional and novel materials towards heavy metal adsorption in wastewater treatment application. J Clean Prod 296:126589. 10.1016/j.jclepro.2021.126589

[CR12] Chen JP, Wu S (2004) Acid/base-treated activated carbons: characterization of functional groups and metal adsorptive properties. Langmuir 20:2233–2242. 10.1021/la0348463 15835676 10.1021/la0348463

[CR13] Dechapanya W, Khamwichit A (2023) Biosorption of aqueous Pb(II) by H_3_PO_4_-activated biochar prepared from palm kernel shells (PKS). Heliyon. 10.1016/j.heliyon.2023.e17250 10.1016/j.heliyon.2023.e17250 PMC10394918 37539182

[CR14] Devault M, Jing L, Arkoun M, Bauerle T, Kessler A, Lehmann J (2025) Biochar shifts balance between hydrophilic and lipophilic molecules in root exudates. Bioresour Technol 427:132426. 10.1016/j.biortech.2025.132426 40120991 10.1016/j.biortech.2025.132426

[CR15] Duque Sistons R, Mayrinck Cupertino GF, Affonso Sampaio D, José dos Santos Júnior A, Dias de Souza N, Francisco Dias Júnior A (2022) Investigating the alkaline treatment of fiber coconut (*Cocos nucifera* L) for cellulosic ethanol generation. Adv Forensic Sci 9:1893–1901. 10.34062/afs.v9i4.13219

[CR16] Dziemianowicz W, Kotarska K, Świerczyńska A (2022) Increase butanol production from corn straw by mineral compounds supplementation. Energies. 10.3390/en15196899

[CR17] Hassaan MA, Yılmaz M, Helal M, El-Nemr A (2023) Isotherm and kinetic investigations of sawdust-based biochar modified by ammonia to remove methylene blue from water. Sci Rep 13:12724. 10.1038/s41598-023-39971-0 37543711 10.1038/s41598-023-39971-0 PMC10404293

[CR18] Ho YS, McKay G (1999) A kinetic study of dye sorption by biosorbent waste product pith. Resour Conserv Recycl 25:171–193. 10.1016/S0921-3449(98)00053-6

[CR19] Huang J, Jiao Y, Weatherley AJ, Duan AX, Wang S, Li C, Ma Z, Liu W, Han B (2023) Catalytic modification of corn straw facilitates the remediation of Cd contaminated water and soil. J Hazard Mater 445:130582. 10.1016/j.jhazmat.2022.130582 37055987 10.1016/j.jhazmat.2022.130582

[CR20] Jiang TJ, Morgan HM, Tsai WT, Chien H, Yen TB, Lee YR (2024) Thermochemical conversion of biomass into biochar: enhancing adsorption kinetics and pore properties for environmental sustainability. Sustainability 16. 10.3390/su16156623

[CR21] Kamdem Tamo A, Doench I, Deffo G, Zambou Jiokeng SL, Doungmo G, Fotsop CG, Tonleu Temgoua RC, Montembault A, Serghei A, Njanja E, Kenfack Tonle I, Osorio-Madrazo A (2025) Lignocellulosic biomass and its main structural polymers as sustainable materials for (bio)sensing applications. J Mater Chem A 13:24185–24253. 10.1039/D5TA02900G

[CR22] Kurzweil P (2025) Supercapacitors | carbon technologies. Encyclopedia of electrochemical power sources. Elsevier, pp 402–425. 10.1016/B978-0-323-96022-9.00046-3

[CR23] Lekrine A, Belaadi A, Dembri I, Jawaid M, Ismail AS, Abdullah MMS, Chai BX, Al-Khawlani A, Ghernaout D (2024) Thermomechanical and structural analysis of green hybrid composites based on polylactic acid/biochar/treated W. filifera palm fibers. J Mater Res Technol 30:9656–9667. 10.1016/j.jmrt.2024.06.033

[CR24] Li R, Tang X, Wu J, Zhang K, Zhang Q, Wang J, Zheng J, Zheng S, Fan J, Zhang W, Li X, Cai S (2023) A sulfonate-functionalized covalent organic framework for record-high adsorption and effective separation of organic dyes. Chem Eng J 464:142706. 10.1016/j.cej.2023.142706

[CR25] Li Z, Wang R, Jia W, Ning P, Cao H (2025) Ionic effect enhanced the adsorption of phosphorus-containing organics (PCOs) from complex wastewater. Chem Eng J 509:161419. 10.1016/j.cej.2025.161419

[CR26] Liu H, Xu G, Li G (2021) Preparation of porous biochar based on pharmaceutical sludge activated by NaOH and its application in the adsorption of tetracycline. J Colloid Interface Sci 587:271–278. 10.1016/j.jcis.2020.12.014 33360900 10.1016/j.jcis.2020.12.014

[CR27] Luo M, Lin H, Li B, Dong Y, He Y, Wang L (2018) A novel modification of lignin on corncob-based biochar to enhance removal of cadmium from water. Bioresour Technol 259:312–318. 10.1016/j.biortech.2018.03.075 29573610 10.1016/j.biortech.2018.03.075

[CR28] Mirițoiu CM, Dobrotă D, Popa D (2025) Designing and study of composites reinforced with shredded corn stalks using a variety of matrices based on dammar, epoxy and acrylic resins. Polym Test 142:142. 10.1016/j.polymertesting.2024.108672

[CR29] Moyo A, Parbhakar-Fox A, Meffre S, Cooke DR (2023) Alkaline industrial wastes – characteristics, environmental risks, and potential for minewaste management. Environ Pollut 323:121292. 10.1016/j.envpol.2023.121292 36804887 10.1016/j.envpol.2023.121292

[CR30] Neme I, Gonfa G, Masi C (2022) Activated carbon from biomass precursors using phosphoric acid: a review. Heliyon 8. 10.1016/j.heliyon.2022.e11940 10.1016/j.heliyon.2022.e11940 PMC9720030 36478849

[CR31] Pathy A, Pokharel P, Chen X, Balasubramanian P, Chang SX (2023) Activation methods increase biochar’s potential for heavy-metal adsorption and environmental remediation: a global meta-analysis. Sci Total Environ 865:161252. 10.1016/j.scitotenv.2022.161252 36587691 10.1016/j.scitotenv.2022.161252

[CR32] Pereira PHF, Maia LS, Silva AIC, Silva BAR, Pinhati FR, Oliveira SA, Rosa DS, Mulinari DR (2024) Prospective life cycle assessment prospective (LCA) of activated carbon production, derived from banana peel waste for methylene blue removal. Adsorption 30:1081–1101. 10.1007/s10450-024-00485-4

[CR33] Premchand P, Demichelis F, Galletti C, Chiaramonti D, Bensaid S, Antunes E, Fino D (2024) Enhancing biochar production: a technical analysis of the combined influence of chemical activation (KOH and NaOH) and pyrolysis atmospheres (N2/CO2) on yields and properties of rice husk-derived biochar. J Environ Manage 370:123034. 10.1016/j.jenvman.2024.123034 39442397 10.1016/j.jenvman.2024.123034

[CR34] Radoor S, Karayil J, Jayakumar A, Parameswaranpillai J, Siengchin S (2021) Efficient removal of methyl orange from aqueous solution using mesoporous ZSM-5 zeolite: synthesis, kinetics and isotherm studies. Colloids Surf A Physicochem Eng Asp 611:125852. 10.1016/j.colsurfa.2020.125852

[CR35] Ray SS, Gusain R, Kumar N (2020) Water purification using various technologies and their advantages and disadvantages. Carbon nanomaterial-based adsorbents for water purification. Elsevier, pp 37–66. 10.1016/B978-0-12-821959-1.00003-9

[CR36] Sahoo SS, Vijay VK, Chandra R, Kumar H (2021) Production and characterization of biochar produced from slow pyrolysis of pigeon pea stalk and bamboo. Clean Eng Technol 3:100101. 10.1016/j.clet.2021.100101

[CR37] Sousa LN, Figueiredo PF, França S, Solar Silva MV, Borges PHR, Bezerra ACS (2022) Effect of non-calcined sugarcane bagasse ash as an alternative precursor on the properties of alkali-activated pastes. Molecules 27:1185. 10.3390/molecules27041185 35208974 10.3390/molecules27041185 PMC8874479

[CR38] Thabede PM, Shooto ND (2025) The removal of methyl orange from water using biochar derived from a blend of carrot and potato peels. Desalin Water Treat. 10.1016/j.dwt.2025.101584

[CR39] Vuong TX, Pham TTH, Nguyen TTT, Pham DTN (2023) Effects of biochar and apatite on chemical forms of lead and zinc in multi-metal-contaminated soil after incubation: a comparison of peanut shell and corn cob biochar. Sustainability 15:11992. 10.3390/su151511992

[CR40] Wang L, Olsen MNP, Moni C, Dieguez-Alonso A, Rosa JM, Stenrød M, Liu X, Mao L (2022) Comparison of properties of biochar produced from different types of lignocellulosic biomass by slow pyrolysis at 600 °C. Appl Energy Combust Sci 12:100090. 10.1016/j.jaecs.2022.100090

[CR41] Wang H, Wang W, Zhou S, Gao X (2023) Adsorption mechanism of Cr(VI) on woody-activated carbons. Heliyon 9. 10.1016/j.heliyon.2023.e13267 10.1016/j.heliyon.2023.e13267 PMC9925964 36798761

[CR42] Wang Q, Xiong J, Song Q, Shaheen SM, Salem HMS, Mohamed I, Majrashi A, Wang S, Rinklebe J, Yao Z, Qi W (2024) Preparation of nitrogen-enriched Fe-doped porous biochar using the catalytic pyrolysis of paper mill sludge. Biomass Convers Biorefin 14:13877–13887. 10.1007/s13399-022-03209-2

[CR43] Wood R, Mašek O, Erastova V (2024) Developing a molecular-level understanding of biochar materials using public characterization data. Cell Rep Phys Sci 5:102036. 10.1016/j.xcrp.2024.102036

[CR44] Xia S, Tao J, Zhao Y, Men Y, Chen C, Hu Y, Zhu G, Chu Y, Yan B, Chen G (2024) Application of waste derived magnetic acid-base bifunctional CoFe/biochar/CaO as an efficient catalyst for biodiesel production from waste cooking oil. Chemosphere 350:141104. 10.1016/j.chemosphere.2023.141104 38171400 10.1016/j.chemosphere.2023.141104

[CR45] Xu Z, He M, Xu X, Cao X, Tsang DCW (2021) Impacts of different activation processes on the carbon stability of biochar for oxidation resistance. Bioresour Technol 338:125555. 10.1016/j.biortech.2021.125555 34303142 10.1016/j.biortech.2021.125555

[CR46] Yi H, Nakabayashi K, Yoon S-H, Miyawaki J (2021) Pressurized physical activation: a simple production method for activated carbon with a highly developed pore structure. Carbon N Y 183:735–742. 10.1016/j.carbon.2021.07.061

[CR47] Yu Q, Zhang X, Gao T, Gong X, Wu J, Tian S, Ma B, Xu L, Joseph S, Zheng J, Bian R, Li L (2024) Converting plastic-contaminated agricultural residues into fit-for-purpose biochar soil amendment: an initial study. Biochar 6:98. 10.1007/s42773-024-00382-7

[CR48] Zeng H, Zeng H, Zhang H, Shahab A, Zhang K, Lu Y, Nabi I, Naseem F, Ullah H (2021) Efficient adsorption of Cr (VI) from aqueous environments by phosphoric acid activated eucalyptus biochar. J Clean Prod 286:124964. 10.1016/j.jclepro.2020.124964

[CR49] Zhang Q, Su C, Tian X, Zhang CY (2023) Corn-based fluorescent light-up biosensors with improved signal-to-background ratio for label-free detection of long noncoding RNAs. Anal Chem 95:8097–8104. 10.1021/acs.analchem.3c01115 37171156 10.1021/acs.analchem.3c01115

